# Evaluating the effects of climate change on US agricultural systems: sensitivity to regional impact and trade expansion scenarios

**DOI:** 10.1088/1748-9326/aac1c2

**Published:** 2018

**Authors:** Justin S Baker, Petr Havlík, Robert Beach, David Leclère, Erwin Schmid, Hugo Valin, Jefferson Cole, Jared Creason, Sara Ohrel, James McFarland

**Affiliations:** 1 RTI International, 3040 East Cornwallis Road, Durham, NC 27709-2194, United States of America; 2 International Institute for Applied Systems Analysis. Schlossplatz 1. A-2361 Laxenburg, Austria; 3 Deptartment of Economics and Social Sciences, University of Natural Resources and Life Sciences. Feistmantelstrasse 4, 1180 Vienna, Austria; 4 United States Environmental Protection Agency, 1200 Pennsylvania Avenue N.W., Washington, DC, 20460, United States of America

**Keywords:** climate change, impacts, agriculture

## Abstract

Agriculture is one of the sectors that is expected to be most significantly impacted by climate change. There has been considerable interest in assessing these impacts and many recent studies investigating agricultural impacts for individual countries and regions using an array of models. However, the great majority of existing studies explore impacts on a country or region of interest without explicitly accounting for impacts on the rest of the world. This approach can bias the results of impact assessments for agriculture given the importance of global trade in this sector. Due to potential impacts on relative competitiveness, international trade, global supply, and prices, the net impacts of climate change on the agricultural sector in each region depend not only on productivity impacts within that region, but on how climate change impacts agricultural productivity throughout the world. In this study, we apply a global model of agriculture and forestry to evaluate climate change impacts on US agriculture with and without accounting for climate change impacts in the rest of the world. In addition, we examine scenarios where trade is expanded to explore the implications for regional allocation of production, trade volumes, and prices. To our knowledge, this is one of the only attempts to explicitly quantify the relative importance of accounting for global climate change when conducting regional assessments of climate change impacts. The results of our analyses reveal substantial differences in estimated impacts on the US agricultural sector when accounting for global impacts vs. US-only impacts, particularly for commodities where the United States has a smaller share of global production. In addition, we find that freer trade can play an important role in helping to buffer regional productivity shocks.

## Introduction

Previous climate change impact assessments of the US agricultural sector have relied in part on domestic partial equilibrium models of the agriculture, forestry, and other land use (AFOLU) sectors (e.g. Beach *et al* 2015, US Department of Agriculture-Office of the Chief Economist USDA-OCE 2013). Although such models often provide substantial detail on US production systems that is valuable for climate impact assessments, these tools typically hold agricultural supply functions fixed in the rest of the world. Thus, even with endogenous trade flows, domestic partial equilibrium models generally ignore potential systemic productivity changes globally under assumed climate change scenarios. A domestic market model can respond to future productivity changes by adjusting consumption patterns and imports and exports of key commodities to reduce the net welfare impacts of the exogenous productivity change. However, this approach can bias results of the climate impact assessment if it does not recognize the effects of climate change on agricultural productivity in the rest of the world and the resulting impacts on relative competitiveness, international trade, and global supply (Costinot *et al* 2016).

This issue is not limited to the United States; many countries have developed country-specific AFOLU baselines and are in the process of conducting impact assessments to develop climate change adaptation plans using country- or region-scale modeling approaches. Country-scale assessments that do not explicitly account for global market interactions or adjust future productivity assumptions in the rest of the world to account for climate change impacts can under- or overproject domestic climate change impacts (Zhang *et al* 2014). In addition, while there have been studies comparing the implications of different trade assumptions across models (Nelson *et al* 2014) and exploring the effects of alternative trade liberalization scenarios (Wiebe *et al* 2015), these studies focus on changes in aggregate global trade rather than from the perspective of individual countries or regions.

To evaluate the potential limitation of country-scale climate impact assessments, this study applies a global model of agriculture and forestry to evaluate climate change impacts on US agriculture with and without accounting for climate change impacts in the rest of the world. To this team’s knowledge, this one of the first attempts to quantify the relative difference in US agricultural sector climate change impacts with and without directly accounting for climate change in the rest of the world, and perhaps the first attempt with a global modeling framework. This goal is accomplished through a scenario design that first isolates several climate change scenarios and exogenous crop yield impacts to the United States only, followed by scenarios that extend the climate impacts to the rest of the world. Then, additional sensitivity analysis is conducted in which global agricultural trade is more rapidly expanded, which provides a buffer against domestic productivity shocks brought on by climate change and opens new possibilities for export of US production. This article focuses on US results to illustrate the potential importance of accounting for global climate impacts when projecting domestic impacts.

## Methods

This analysis applies the Global Biosphere Management Model (GLOBIOM), a detailed partial equilibrium model of the global agriculture, forestry, and bioenergy sectors. GLOBIOM represents the world partitioned into 30 economic regions^6^, in which a representative regional consumer optimizes their consumption, depending on income, preferences, and product prices. On the production side, producers maximize their margins, and the model solves for a market equilibrium corresponding to the overall welfare maximization based on the spatial equilibrium modeling approach (Takayama and Judge 1971, McCarl and Spreen 1980). Additional information on the model structure and key parameters can be found in (Havlík *et al* 2011, Havlík *et al* 2014).

The supply side of the model relies on spatially explicit information based on the concept of simulation units, which are aggregates of 5 pixels belonging to the same altitude, slope, and soil class, within the same 30 arcmin pixel and within the same country. For crops, livestock, and forest activities, production systems’ parameters are built using detailed biophysical models such as EPIC for crops, RUMINANT for livestock (Herrero *et al* 2013), and G4M for forestry (Forsell *et al* 2016). For this study, the supply-side spatial resolution is aggregated to 120 arcmin (approximately 200 × 200 km at the equator). The model is calibrated to historical FAOSTAT activity data representing the year 2000, including production levels and prices. The model baseline and all subsequent climate change scenarios are recursively solved in 10 year time steps until 2050. Comprehensive greenhouse gas accounting for agriculture, forestry, and other land use is implemented in the model. Detailed descriptions of these accounts and additional background information are provided in Valin *et al* (2013) and Havlík *et al* (2014).

This study uses global gridded yield impacts available from the Inter-Sectoral Impact Model Intercomparison Project (ISI-MIP) archive (www.isimip.org/) that were estimated using EPIC, as further described in Leclère *et al* (2014), for our primary scenarios. Scenarios are developed for all representative concentration pathways (RCPs) and general circulation model (GCM) combinations included in the ISIMIP archive. Additional detail on climate impact assumptions as well as sensitivity analyses conducted is provided in the supplemental appendix available at stacks.iop.org/ERL/13/064019/mmedia.

### Scenario design

Table 1 summarizes the RCP-GCM combinations assessed for this study. We analyze climate impacts under each of the four representative concentration pathways (RCPs) (van Vuuren *et al* 2011) as simulated by the HadGEM2-ES general circulation model (GCM) (Collins *et al* 2011, Martin *et al* 2011) to represent impacts under a range of climate outcomes. In addition to HadGEM2-ES, we conduct analyses for four additional GCMs for the high climate forcing RCP8.5 scenario to reflect variation across climate models: GFDL-ESM2M (Dunne *et al* 2012), IPSL-CM5A-LR (Dufresne *et al* 2013), MIROC-ESM-CHEM (Watanabe *et al* 2010), and NorESM1 M (Bentsen *et al* 2013), as presented in table 1. The HadGEM2-ES model is simulated with and without the effects of CO_2_ fertilization under RCP8.5 to further explore the implications of this effect. Each of these RCP and GCM combinations (denoted in the table by X) are analyzed using GLOBIOM, first considering domestic climate impacts only, and then incorporating climate impacts on all regions of the world.

For domestic zone of impact scenarios, exogenous productivity changes are only applied to US production systems; thus, agricultural yields in the rest of the world evolve over the simulation horizon consistent with baseline assumptions (although changes in production patterns globally can result in endogenous yield changes outside of the United States). These domestic impacts scenarios account for global trade and market interactions, but the US region is the only modeled region that experiences future climate shocks to crop and grassland productivity. Conversely, the global zone of impact climate scenarios apply the exogenous productivity shocks broadly across all regions in the model. By focusing on impacts to US production systems (e.g. production, consumption, and prices), this scenario design allows us to isolate the impact of moving from a domestic-only impacts case to one in which projected climate-induced productivity changes are present in all regions. Comparing results across these two sets of scenarios reveals important differences in domestic (US) crop production trends and key market outputs. To model this option, we include a scenario in which the increasing trade cost element of the GLOBIOM trade specification, which represents the hurdle to substantially expand trade in a given period because of limited infrastructure, non-tariff trade barriers, regional preferences, and other factors, is reduced to almost zero.

Finally, we evaluate the robustness of our results through sensitivity analysis around crop model inputs and alternative trade assumptions. Specifically, we incorporate exogenous crop yield projections using the Lund-Potsdam-Jena managed Land (LPJmL) crop model and assess intensive margin trade expansion through reduced tariff scenarios as adopted in some other studies such as Wiebe *et al* (2015). Full details on the scenario design and results from these robustness checks are summarized in the supplement.

## Results and discussion

In our presentation of results, we focus on projected changes relative to the baseline for major US crops (barley, corn, cotton, rice, sorghum, soybeans, and wheat), livestock commodities (ruminant meats, nonruminant meats, and poultry), and agricultural land uses (cropland and grasslands). The results and discussion provided below focus on the economic responses to exogenous climate shocks, presenting endogenous changes to relevant production and market outputs across climate scenarios as landowners respond to changes in the relative profitability of alternative land uses and consumers respond to changes in commodity availability and prices. Additional discussion and figures summarizing the exogenous yield changes used as inputs to GLOBIOM are provided in the supplemental materials.

### Changes in agricultural outputs across climate scenarios

Once exogenous productivity changes have been introduced into the model, scenario simulations are performed that are designed to maximize total economic surplus over decadal time steps (beginning in 2010 and ending in 2050). Markets and regional production and consumption patterns adjust to the assumed yield and trade conditions. The results presented below focus on impacts on US agricultural systems across the RCP-GCM combinations simulated with only domestic climate impacts (USA) and with climate impacts on all regions of the world (WLD). We also examine scenarios with base GLOBIOM trade assumptions (T0) and liberalized trade (T2).

**Figure 1.**
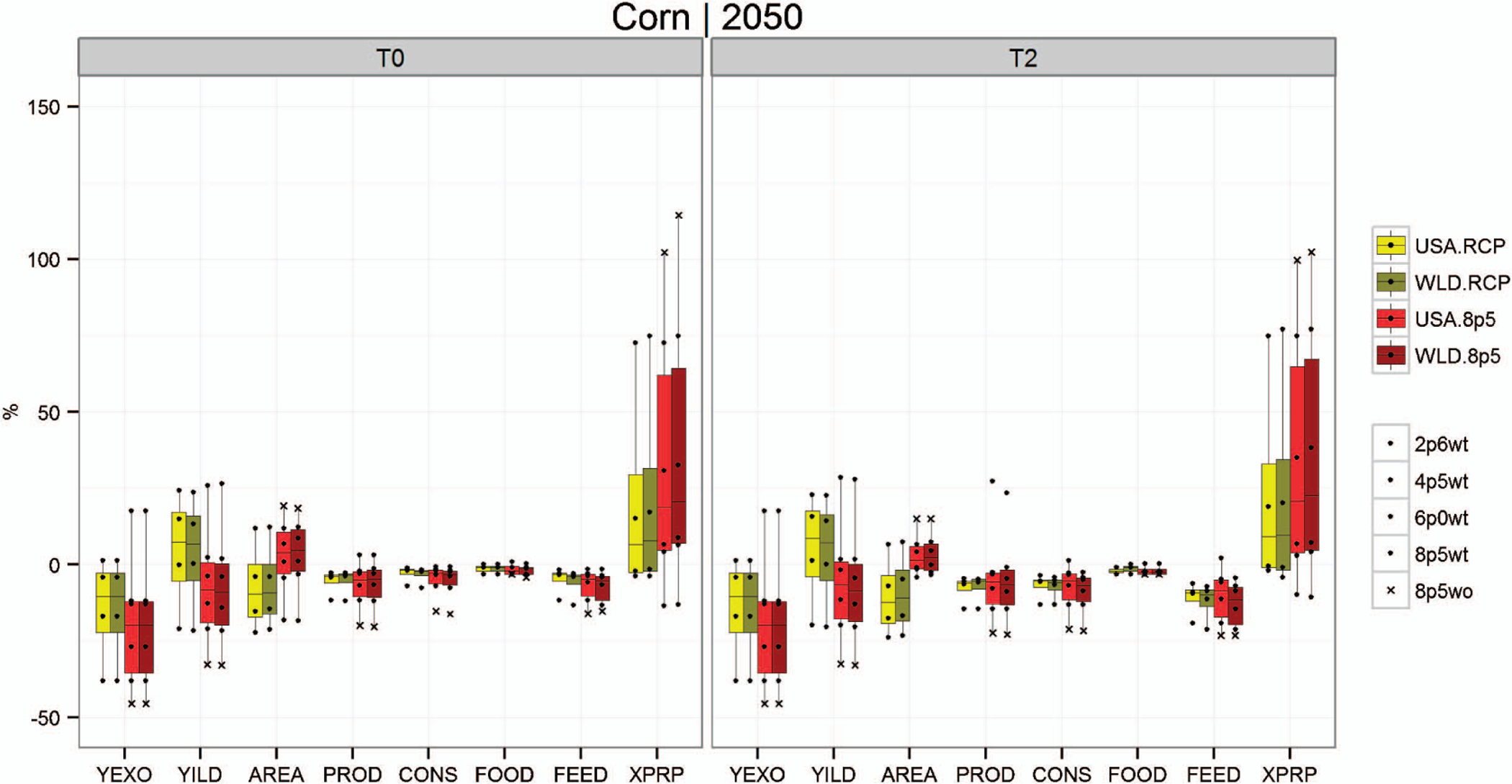
Projected climate impacts for US corn production systems across RCPs and GCMs when climate impacts are applied only domestically (USA) vs. climate impacts applied to the entire world (WLD). Values are provided for changes in exogenous yield inputs (YEXO), endogenous yield aftermarket responses (YILD), crop area (AREA), production (PROD), total consumption (CONS) along with subcategories reflecting consumption for food (FOOD) and feed (FEED), and prices (XPRP). Yellow shaded bars show the interquartile range across all four RCPs with CO_2_ fertilization for the scenarios where climate impacts are applied only domestically (USA). Green shaded bars show the interquartile range for the four RCPs with CO_2_ fertilization for the scenarios where climate impacts are applied globally (WLD). Red bars show the interquartile range across five GCMs for RCP8.5 (with [8p5wt], and for HadGEM-ES2 also without [8p5wo] CO_2_ fertilization) for domestic-only impacts scenarios. Dark red bars show the interquartile range for GCMs for RCP8.5 (with and without CO_2_ fertilization) for global impacts scenarios. Both baseline trade assumptions (T0) and expanded trade (T2) are shown.

**Figure 2.**
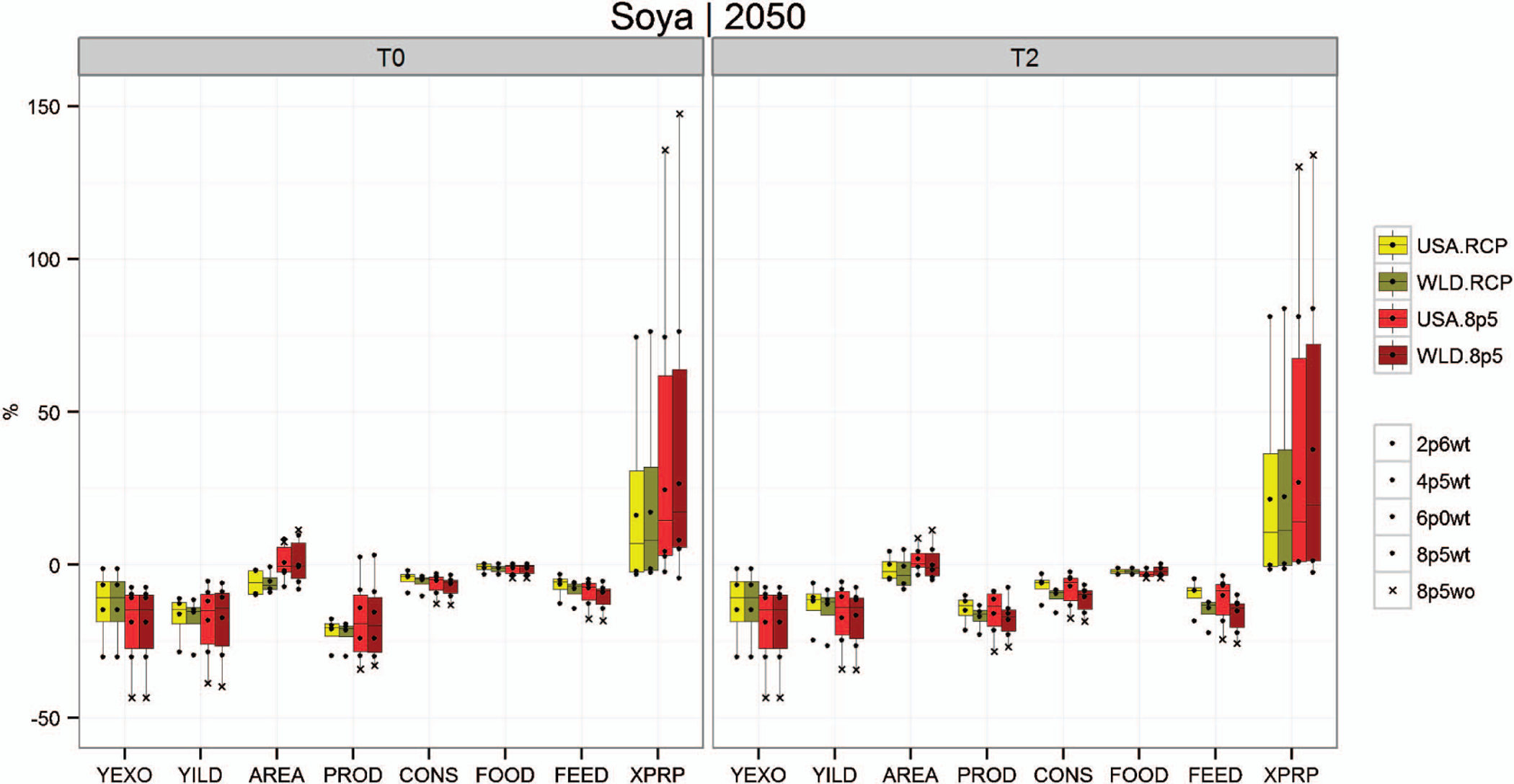
Projected climate impacts for US soybean production systems across RCPs and GCMs when climate impacts are applied only domestically (USA) vs. climate impacts applied to the entire world (WLD).

**Figure 3.**
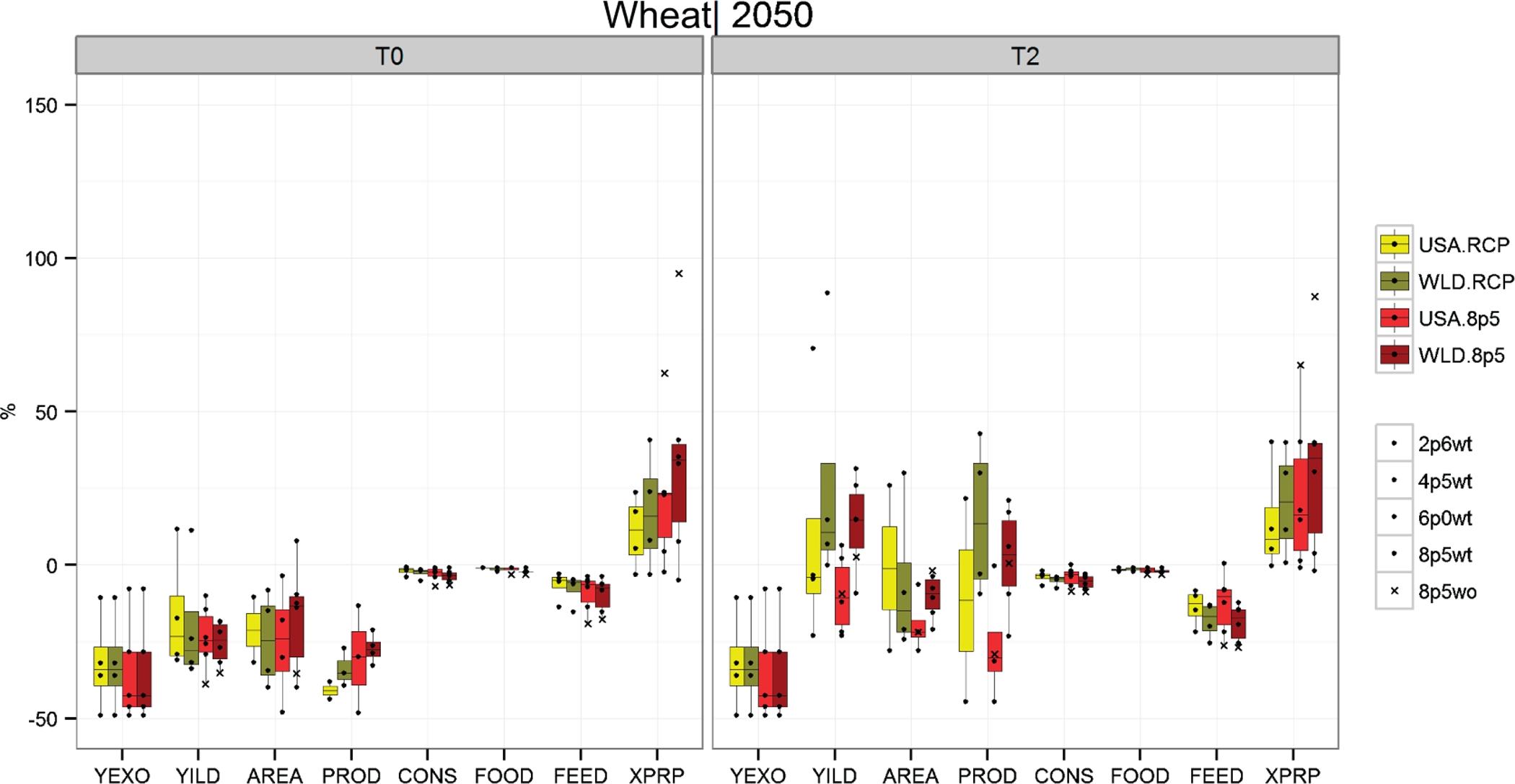
Projected climate impacts for US corn production systems across RCPs and GCMs when climate impacts are applied only domestically (USA) vs. climate impacts applied to the entire world (WLD).

Figures 1–3 show box plots that illustrate the projected range in relevant crop outputs across the various simulation scenarios. The range captured by the box plots conveys the differences in projected model outputs across five GCMs and four RCPs. At the far left of each figure is the endogenous yield response (YILD) to the climate change scenarios, which is obtained from the GLOBIOM model solution. These endogenous yield projections include the simulated crop yield response from the EPIC model (YEXO) and the endogenous land management responses from the GLOBIOM solution. These responses include both shifts to the intensive (shifting to higher input production systems or irrigated systems) and extensive crop production margins (land use and crop mix changes).

The remaining variables represented in figures 1–3 include crop area (AREA), production (PROD), total consumption (CONS) along with subcategories consumption for food (FOOD) and consumption for feed (FEED), and prices (XPRP). Lighter colors represent impacts for the US-only climate change scenarios (USA), and the darker bars represent scenarios where climate impacts are incorporated globally (WLD). Yellow- and green-shaded box plots show the distribution of outcomes across RCPs (using the HadGEM2-ES GCM projections), including RCP 8.5 with CO_2_ fertilization. The red bars show the distribution across GCMs for RCP 8.5 with CO_2_ fertilization for all scenarios as well as without CO_2_ fertilization for HadGEM2-ES. These figures help illustrate the magnitude and potential variability of impacts on the US agricultural sector across multiple domains (GCM, RCP, with and without CO_2_ fertilization, with and without global impacts [USA and WLD] and with and without increased trade). Table 2 provides average impacts across RCPs and GCMs for a larger set of crop groups.

Figure 1 focuses on net impacts for US corn production, consumption and prices by 2050 and relative to a no climate change baseline. For the RCPs with less severe impacts (2.6–8.5 with CO_2_ fertilization) net impacts vary significantly. Assuming base trade assumptions, net (endogenous) yield changes (YILD) range from highly positive (more than 25%) to negative (approximately −25%) when moving from RCP 2.6 to RCP 8.5. For RCP 8.5, the distribution of yield is primarily negative, but the range across GCMs for RCP 8.5 is larger than the range across RCPs for the HadGEM2-ES GCM. Under RCP 8.5, one scenario results in a positive change in endogenous yield, while all other scenarios are negative, ranging from −5% to −35%. The implied variability range across GCMs for RCP 8.5 is greater for variables related to corn production systems and markets than the implied variability range for RCPs 2.6–8.5 with CO_2_ fertilization.

Total corn production shows a smaller net change (a decrease of less than 15% for RCPs 2.6 through 8.5 with CO_2_ fertilization and a range of −20% to 5% for RCP 8.5 across all GCMs). Corn area declines under RCPs 2.6 through 8.5 for HadGEM2-ES, and increases for most RCP 8.5 scenarios, including a change of more than 20% when CO_2_ fertilization is not included. Although corn area increases in some scenarios, total corn production declines in almost all scenarios relative to the no climate change baseline. This change in production results in a net decrease in corn consumption, most of which comes from feed use. This shift is partially driven by improved productivity and utilization of grasslands for livestock feeding as a substitute for traditional feed grains. Feed grain markets absorb the greatest share of the consumption impact because the proportionate change in consumption of corn for feed is higher than consumption for food. Corn price impacts induced by production and consumption changes are substantial and positive for most scenarios. Furthermore, corn price impacts vary significantly relative to the no climate change baseline, ranging from −12% to more than 100%.

Figure 2 shows impacts on US soybeans by 2050 relative to the no climate change baseline. Projected impacts include negative endogenous yield changes across all scenarios and total area impacts ranging from slightly negative to slightly positive (−10% to 10%). These yield and soybean area changes reduce total production, with an average production decline of approximately 18.5% across all scenarios. Net consumption decreases relative to the baseline in all scenarios, with most of this impact felt in the feed market. Prices see substantial increases with large variability, averaging approximately 25% across all scenarios. Across RCPs, price impacts range from slightly negative to approximately 75% higher than the no climate change baseline. Across GCMs for RCP 8.5, price impacts range from slightly negative to more than 125%. Similar to corn, the implied variability range across GCMs for RCP 8.5 is greater for soybean simulation outputs than for across RCPs.

US wheat production systems see a larger endogenous yield impact relative to corn and soybeans (figure 3). Wheat yields decline approximately 20% to 30%, on average, relative to the baseline for all scenarios. Unlike corn and soybeans, however, total wheat area declines significantly across most scenarios, with an average contraction of approximately 25%. Lower yields and reduced area correspond to a substantial decrease in total US wheat production, which averages more than 35% for all scenarios. This change in wheat production is significant, especially when compared with corn and soybeans; however, projected wheat consumption impacts are relatively modest (close to zero). Also, wheat price impacts are less severe than for corn and soybeans. Across RCPs, price impacts are less than 25% on average. Across RCP 8.5 scenarios, prices increase by less than 40% on average. Thus, even with larger declines in total wheat area and production relative to other staple crops, wheat markets are relatively more stable than corn and soybeans, resulting in less price response to the climate impacts scenarios.

Changes in crop and livestock production result in large projected land use changes relative to the climate change baseline. Table 3 shows these net changes in US cropland and grassland areas (extensive margin response) across different climate scenarios. Improved productivity on US grasslands (intensive margin response) has a meaningful effect on land use change. US grassland utilization increases 4.8%–11.3% across all climate change scenarios in the 2050 simulation period relative to the baseline. This increase is accompanied by a similar decline in cropland use. Total US cropland declines 4.4%–16.5% by 2050 relative to the no climate change baseline, and the extent of this decrease is negatively correlated to the magnitude of the projected exogenous yield impacts for the climate change scenarios. For example, RCP 2.6 shows relatively modest or slightly increasing yields, so total US cropland area sees the greatest net contraction for this scenario. Other things being equal, higher US yields mean that less cropland is needed to meet the demand for agricultural commodities.

Exogenous yield impacts on crops are most negative under RCP 8.5 without CO_2_ fertilization, which tends to drive up agricultural commodity prices and increases the opportunity costs of moving land out of crop production and into grassland for grazing purposes. However, if this scenario is excluded from the assessment, we find that reductions in total cropland utilization are smaller for RCPs 2.6–8.5, averaging −12.9% and 10.3% for the USA and WLD zone of impact scenarios, respectively. While cropland declines across all scenarios examined, grassland expands as feeding operations shift from grain based to forage based to accommodate reduced crop productivity and higher grassland yields. For RCP 8.5 scenarios with CO_2_ fertilization, the contraction in cropland is smaller on average, and the expansion in pasture is greater on average than for RCPs 2.6–8.5, indicating a general extensive margin effect present under RCP 8.5 driven by lower overall yields. More information on livestock sector impacts across these simulation scenarios can be found in the online supplement.

### Comparing domestic and global climate impacts scenarios

**Figure 4.**
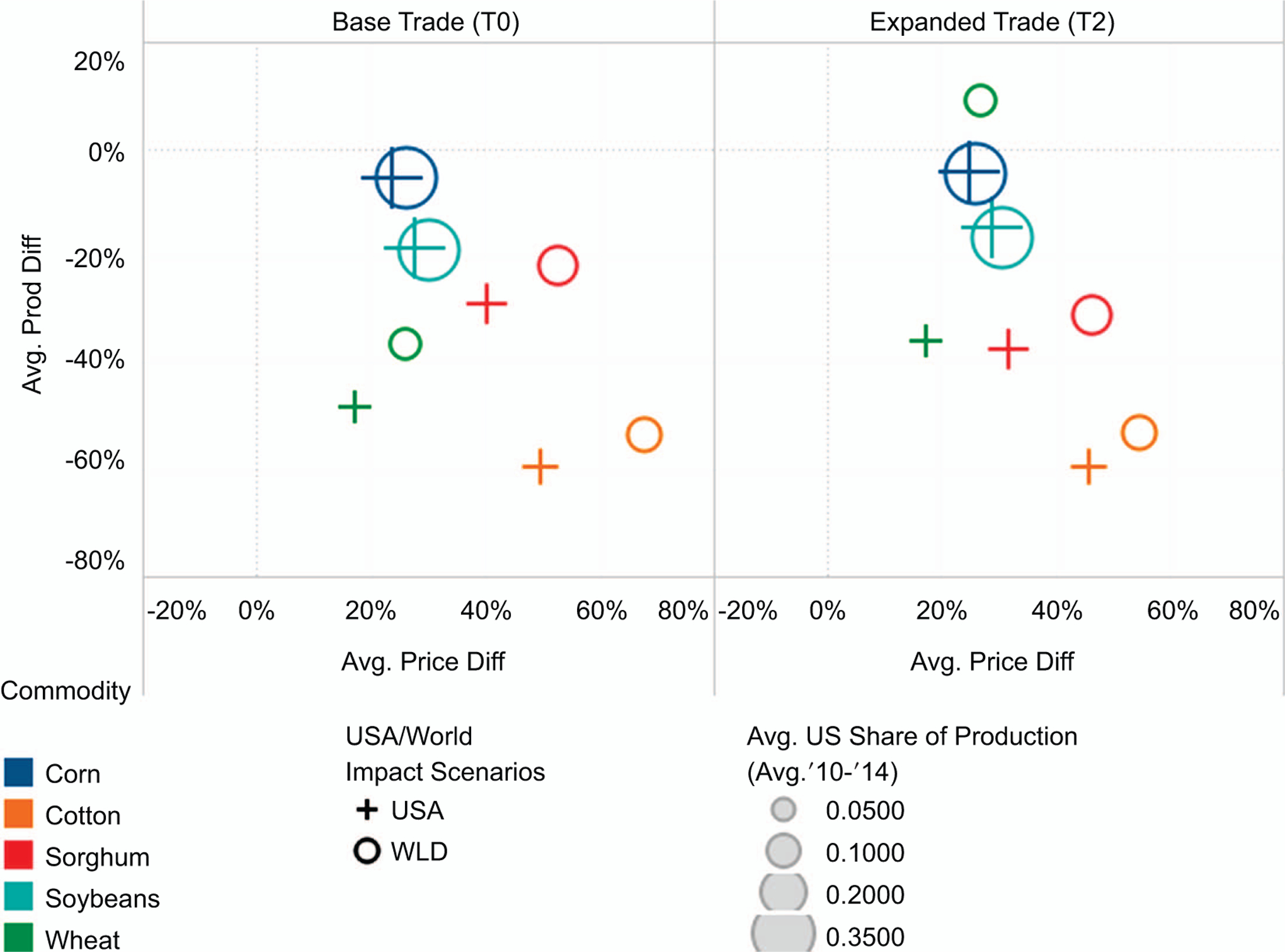
Average production and price impacts in the US for key crop commodities across RCP and GCM scenarios and relative to recent US market share. The left panel figure includes average impacts for the scenarios with base model trade assumptions (T0) and the right-hand side shows average impacts with expanded trade (T2). Domestic (USA) and global climate impacts scenarios (WLD) are differentiated by symbol. The size of each shape corresponds to a metric related to market power. The size of the symbol corresponds to the US share of observed global production from 2010–2014 for the specific commodity group.

When shifting from domestic to global climate change impacts scenarios, the net difference in impacts varies greatly across commodity groups. Figure 4 shows the average impact on prices and production for different crop commodities (averaging over all RCP scenarios, and percentage differences are calculated relative to the baseline). Figure A1 in the supplemental appendix conveys the same information, only for livestock commodities.

For some crop commodities, such as corn and soybeans, the implied change when moving from domestic to global impacts scenarios is relatively small, though even small percentage changes relative to baseline can lead to sizable changes in producer and consumer welfare when examining large markets such as these. Projected corn and soybean prices do increase under the global scenarios relative to the domestic impacts scenarios, but this relative shift is less than 10% for both commodity groups (figure 4). The net corn price impact relative to the no climate change baseline increases from 23.6%–26.2%, while the net impact on soybean prices increases from 27.8%–30.3%. Production impacts are even less pronounced, with the average change in projected corn production shifting from −5.6% to −5.5% and the change in soybean production shifting from −18.5% to −18.8%. With these relatively small responses in total production, most of the net change in prices for corn and soybeans can be attributed to systemic productivity declines outside of the United States under the global scenarios. Lower global productivity results in higher prices generally, although this does not result in large changes in US corn or soybean production.

For other crops, however, the shift from domestic to global climate change scenarios is more pronounced. As figure 4 shows, projected production and price impacts for cotton, sorghum, and wheat show larger differences when comparing domestic-only to global climate impacts scenarios. The projected US wheat production impact changes from −48.0 to −36.4% in 2050, while the net price increase shifts from 17.1 to 26.2%. Unlike corn and soybeans, the net change in US crop area and total production is smaller under global climate change scenarios than for domestic impacts scenarios.

The primary reason for the difference in the directionality and relative magnitude of these impact shifts for wheat and other commodity groups is because the United States holds a smaller global market share for these commodities than for corn or soybeans. The United States accounted for roughly 34% of global production of both corn and soybeans and 36% and 43% of global exports, respectively, from 2010–2014 (Food and Agriculture Organization 2016). Conversely, US wheat production only accounted for approximately 8.4% of global production and 18% of global exports during the same time period. US cotton commands less than 20% of the global share of both production and exports, and while US sorghum controls almost one-half of the global export share, the US production share is less than 20%.

Thus, crop commodities with relatively smaller global market shares see larger net changes in projected climate impacts when moving from domestic to global climate impacts scenarios. This result is intuitive considering that a higher total share of production and exports for a crop group would imply a greater net effect on world prices given local climate change impacts, regardless of production shifts in the rest of the world. That is, any significant change to US corn and soybean production systems would have important implications for global market effects, leading to higher overall price impacts.

The global wheat market is less reliant on US wheat production overall, so even a large shift in US production under the US-only climate scenarios does not result in similarly scaled price impacts. Commanding a smaller market share globally offers greater flexibility for US wheat producers and consumers to adapt to lower yields induced by climate change by shifting production and export patterns. When global climate impacts are accounted for, however, the difference in net price impacts for US wheat is much larger (approximately 50% higher than the projected price impact under the US-only RCP 8.5 scenario). The net decrease in wheat area and total production are smaller under the global climate change scenarios, because US wheat production reacts to climate-induced productivity changes in the rest of the world. Sorghum is a bit of an outlier in that the US export share is high, but the absolute value of US exports is minimal relative to corn. Furthermore, because sorghum is highly fungible with corn in feed and other markets, it is reasonable to see a larger relative change in projected impacts across scenarios when corn production systems are relativelys table.

To evaluate whether there is a statistically significant difference between projected mean impacts for domestic and global zone of impact scenarios, we ran a simple analysis of variation (ANOVA) on percentage differences from baseline for different variables and across crop and livestock commodity groups. Trade scenarios were controlled for using indicator variables. Consistent with the data visuals, we found low statistical significance for crop groups such as corn and soybeans, but high significance for wheat (among other commodity groups). It is important to note, however, that standard statistical tests are limited in this context as we are comparing results from a relatively small number of discrete model simulations that assume different input parameter values that are not pulled randomly from a common distribution as in the case of Monte Carlo analysis or equivalent stochastic framework.

### Implications of trade scenarios

Reducing barriers to trade expansion dampens net climate change impacts on commodity markets by reallocating resources and production patterns globally based on comparative advantages to a greater extent. These shifts typically result in reduced price impacts and increased supply globally, and this result is consistent with findings in other recent studies (Leclère *et al* 2014). Although this finding is important, the following discussion focuses on the interaction between trade expansion and the regional climate impacts scenario design.

For some commodities, projected US impacts are not significantly affected by trade scenarios. For example, corn and soybean price and production impacts under expanded trade are very close to those with base model trade assumptions. The most noticeable change for corn and soybeans is a slight decrease in the average US production impact under T2 scenarios (that is, more net production with expanded trade), although projected price impacts are approximately the same. For other crop and livestock commodity groups, this result does not hold, and production and price effects vary greatly. For example, US sorghum shows greater relative declines in total production relative to the base trade assumptions (table 2), but price impacts are smaller because global production systems have greater flexibility to adjust to changing climate regimes. Average production impacts for US cotton are similar across the two trade scenarios, but the net change in the price effect for cotton between the domestic and global scenarios is smaller with expanded trade.

The most meaningful difference occurs for projected wheat impacts. Under the T2 scenarios, average wheat price impacts are quite similar to average price impacts under the T0 scenarios. However, US wheat production impacts shift from an average change of −36% under the local climate impacts scenarios to +9.1%. Furthermore, the variability range around wheat production, area, and yield impacts relative to the baseline is much larger with expanded trade than under base trade assumptions. Thus, relaxing trade restrictions amplifies both the relative impact of moving from domestic-only to global climate impacts scenarios for US wheat and introduces a new source of variation in projected impacts captured by the interaction between trade dynamics and climate change impact assumptions. On average, however, expanded trade possibilities shift global production to the United States because it holds a comparative advantage in wheat production. The United States captures a greater total market share of global wheat production and exports over time.

Livestock sector production mostly declines under the expanded trade scenarios relative to base trade assumptions. Similar to crops, net price impacts are smaller with expanded trade. US production of bovine meat increases under expanded trade, although the relative difference in impacts between the domestic and global scenarios does not change. Note that under both the base trade and expanded trade cases, US grassland utilization and beef production are higher under the domestic impacts scenarios, and this effect is marginally higher under the global scenarios.

## Conclusion

In summary, this analysis presents a new assessment of climate change impacts on the US agricultural sector using a detailed global model of the land use and bioenergy sectors (GLOBIOM). Results show meaningful potential impacts on crop productivity, total production, and prices by 2050 across assumed climate scenarios (which were developed based on the ISIMIP archives). Net impacts increase with the assumed RCP; production and price impacts are significantly higher under RCP 8.5, including large price effects for key commodity groups such as corn and soybeans.

Our scenario design enables us to directly evaluate the importance of accounting for global climate change impacts on US agricultural outputs relative to a scenario in which US impacts are evaluated in isolation (holding climate constant in the rest of the world). While the net impact of moving from local to global climate scenarios is relatively small for crop and livestock commodity groups for which the United States commands a dominant global market share, the changes are substantial for commodities that maintain a smaller total share of global production and exports. Further research is needed to assess whether this relationship holds for other regions and commodity groups, especially for regions that are heavily dependent on agricultural imports with growing food security concerns.

With respect to trade responsiveness to climate change impacts, the model intercomparison presented in Nelson *et al* (2014), note that GLOBIOM is consistent with models that assume greater market integration by 2050 in the default setup relative to other models. From this perspective, and in particular for the increased market integration scenario (T2), our study provides a kind of boundary of the magnitude of external climate change impacts on the US market. The weaker the assumed future market integration, the less important it would be to consider the effects of external climate change impacts on domestic markets.

Future climate impacts analyses of country-scale agriculture systems could benefit from additional consideration of global impacts and trade adjustments. Failure to do so could result in understated price impacts and a significantly different future crop mix and agricultural management profile than a future projection that accounts for climate impacts globally. In conducting impact assessments or developing agricultural sector adaptation plans, countries should account for changes in global supply and markets under assumed climate change scenarios and how this might influence country-level production and trade decisions. Ignoring global effects in local climate impact assessments can over- or underestimate the net effects of climate change, particularly for commodities in which a country holds a relatively small total market share. However, robustness checks using an alternative crop model and summarized in the supplement show that the potential bias resulting from ignoring global climate impacts and connections to the global market system likely decreases if projected yield changes under alternative climate scenarios are positive.

Finally, the results from this study show benefits to extensive margin trade expansion in terms of reduced climate change impacts, especially for the global climate change scenarios. Reduced tariff scenarios, summarized in the supplement, can also reduce the magnitude of key impact metrics. These findings suggest that country-level climate adaptation plans should consider reducing trade barriers in addition to investments that improve the resilience of agricultural systems.

## Supplementary Material

supplement

## Figures and Tables

**Table 1.** RCP and GCM scenario combinations run for this analysis. The HadGEM2-ES model was simulated both with and without CO_2_ fertilization under RCP8.5, yielding a total of 9 combinations analyzed for each set of regional impact and trade assumptions.

	RCP 2.6	RCP 4.5	RCP 6.0	RCP 8.5
**HadGEM2-ES**	X	X	X	X
**GFDL**				X
**IPSL**				X
**MIROC**				X
**NORESM**				X

**Table 2.** Average crop production and price impacts for the US relative to the no climate change baseline across RCP and GCM scenarios (2050 simulation period). Changes in USA production (top) and price (bottom) for major agricultural commodities are presented for scenarios where climate impacts are applied only to the USA (USA, on the left of each of the four sets of results) and to the entire world (WLD).

Production impacts				
Trade	Base trade (T0)		Expanded trade (T2)	
Regional extent of climate impacts	USA	WLD	USA	WLD
**Barley**	−11.5%	−9.9%	−25.7%	−19.5%
**Corn**	−5.6%	−5.5%	−4.4%	−4.9%
**Cotton**	−59.7%	−53.6%	−59.4%	−53.1%
**Rice**	−63.9%	−61.3%	−76.1%	−73.4%
**Soybeans**	−18.5%	−18.8%	−14.6%	−16.8%
**Sorghum**	−28.9%	−22.0%	−37.4%	−31.3%.
**Wheat**	−48.3%	−36.4%	−36.1%	9.1%
	Price impacts			
Trade	Base trade (T0)		Expanded trade (T2)	
Regional extent of climate impacts	USA	WLD	USA	WLD
**Barley**	25.5%	28.5%	28.3%	31.5%
**Corn**	23.6%	26.2%	25.1%	25.9%
**Cotton**	49.8%	67.8%	45.8%	54.7%
**Rice**	2.4%	3.1%	1.0%	1.6%
**Soybeans**	27.8%	30.3%	29.0%	30.8%
**Sorghum**	40.3%	52.7%	31.7%	46.4%
**Wheat**	17.1%	26.2%	17.2%	26.8%

**Table 3.** Changes in average cropland and grassland areas for the US relative to the no climate change baseline across RCP and GCM scenarios (2050 simulation period and with base trade assumptions). Results are presented for scenarios where climate impacts are applied only to the USA (USA) and to the entire world (WLD).

	Cropland area		Grassland area	
Regional extent of climate impacts	USA	WLD	USA	WLD
**2p6wt_HadGEM**	−16.5%	−12.9%	7.7%	6.9%
**4p5wt_HadGEM**	−11.3%	−10.0%	11.3%	10.7%
**6p0wt_HadGEM**	−14.6%	−13.7%	8.1%	7.5%
**8p5wt_GFDL**	−4.4%	−4.4%	10.7%	10.3%
**8p5wt_HadGEM**	−9.2%	−7.5%	7.5%	6.9%
**8p5wo_HadGEM**	−5.2%	−3.2%	4.8%	4.4%
**8p5wt_IPSL**	−4.7%	−3.6%	9.0%	7.9%.
**8p5wt_MIROC**	−6.6%	−2.6%	9.0%	8.2%
**8p5wt_NorESM**	−5.2%	−2.5%	8.1%	7.0%
**Average**	−8.6%	−6.7%	8.5%	7.8%

## Footnotes

6 This analysis aggregates regions and provides results at an 11-region level.
