# Supplementary material for: Evaluating the effects of climate change on US agricultural systems: sensitivity to regional impact and trade expansion scenarios
Source: Environ Res Lett. Author manuscript; Available in PMC 2020 Mar 9. (PMC7061454; doi:10.1088/1748-9326/aac1c2)
Supplement: supplement [file NIHMS1563902-supplement-supplement.pdf]

SUPPLEMENTAL MATERIAL

This supplement provides additional information on several elements of this analysis, including:

1) Scenario design details
2) More information on the GLOBIOM framework, including two components critical to this analysis:
    - exogenous crop yield impacts developed by the EPIC global crop model that are incorporated into the model as scenario inputs, and
    - additional information on the GLOBIOM trade specification.
3) Results and discussion around livestock sector impacts
4) Description of scenario design and results and discussion around robustness check scenarios, including:
    - Additional scenarios that use exogenous crop yield changes from the LPJmL crop model,
    - Alternative trade scenarios that reduce intensive margin trade costs (tariffs).

Scenario Design Details

In addition to the nine climate change impact scenarios outlined in Table 1 (and elaborated on in Supplemental Material), we develop a scenario design that enables us to examine the relative importance of accounting for global climate change impacts when projecting the potential sensitivity of the US agricultural production system to climate change.

The scenarios capture variability in projected impacts across RCPs for a single general circulation model[1] (GCM), HadGEM2-ES (Collins et at 2011, Martin et al 2011) in this case, as well as variability across GCMs for a single RCP (8.5, evaluated with and without CO_2 fertilization). While there has long been recognition in the literature that CO_2 fertilization could have meaningful impacts on crop yields, there remains considerable uncertainty on the extent of this effect, so we include the RCP 8.5 without fertilization scenario in the analysis as an upper bound on exogenous yield impacts. Furthermore, not including CO_2 fertilization is consistent with numerous previous studies such as an evaluation of impacts on food security (Nelson et al., 2010), the AgMIP modeling efforts (Nelson et al., 2014), and other recent studies (Hasegawa et al., 2016; Dellink et al., 2017)[2], which allows one to evaluate our high impact scenario results relative to previously published impacts projections that did not consider CO_2 fertilization.

Furthermore, there is a growing literature that explores the uncertainty in physical estimates of yield impacts across various crop models (e.g., Asseng et al., 2013, Bassu et al., 2014; Li et al., 2014; Rosenzweig et al., 2014; Fleisher et al., 2017). Therefore, we also complement the analysis relying on the GLOBIOM “native” crop growth model EPIC by a sensitivity test using the LPJmL model (Bondeau et al. 2007), which according to the ISI-MIP crop growth model intercomparison represents the more optimistic models, while EPIC being more pessimistic in the yield impacts projections (Rosenzweig et al. 2014).

[1] General circulation models (GCMs) are a type of climate model. GCMs are numerical models that represent physical processes in the atmosphere, ocean, cryosphere, and land surface used to model global climate systems.
[2] There are two primary approaches for estimating climate change impacts on yields: process-based crop simulation models and statistical models. While process-based crop simulation models are often applied with and without CO_2 fertilization, yield impacts estimated using statistical models typically do not include CO_2 fertilization due to difficulties in statistical estimation of this effect (Lobell and Asseng, 2017). Thus, the majority of economic studies relying on inputs derived from statistically estimated models as well as many using process-based models (e.g., Nelson et al., 2014) do not reflect CO_2 fertilization effects.

For trade integration (T2) scenarios, all baseline trade cost elasticities in GLOBIOM are multiplied by 20, and the slope of the quadratic function is divided by four. This approach stimulates extensive margin trade expansion, incl. new trade flows and also brings the GLOBIOM trade cost representation close to other partial equilibrium models that consider the trade cost independent from traded quantity. Additional information on the GLOBIOM trade specification can be found in the Supplemental Material.

GLOBIOM Model Details—EPIC Projections and Trade Specification

EPIC Exogenous Crop Yield Impacts:

GLOBIOM is combined with the EPIC crop model (Williams 1995) to calculate the impact of climate change on the agricultural sector, following methods described in (Leclère *et al* 2014) and (Havlík *et al* 2015). EPIC is used to simulate yield associated with each location, management practice, and climatic conditions. The model estimates the biophysical and environmental parameters of 18 crops for three different types of management systems (low input rain-fed, high input rain-fed, and irrigated systems). To project impacts of climate change, daily climate input data of solar radiation, min and max temperature, precipitation, relative humidity, and wind speed are taken as inputs from the climate models, as well as future atmospheric CO_2. Because the net CO_2 fertilization effect is still debated in the literature (Tubiello *et al* 2007), this analysis considers two different levels of CO_2 fertilization responses—full and none. EPIC simulations include some adjustments in crop management intensity in response to climate, such as marginal changes to fertilizer and irrigation water use, as well as shifts in annual planting and harvesting dates. Other larger scale adjustments (e.g. management intensification from high input rain-fed to high input irrigated) are endogenously determined by GLOBIOM.

To reflect potential variability in future greenhouse gas emissions and associated climate change, our scenario design includes four RCPs with bias-corrected climate projections from the HadGEM2-ES global circulation model (RCP 2.6, RCP 4.5, RCP 6.0, and RCP 8.5). For RCP 8.5, climate projections from other GCMs are also considered (IPSL-CMSA-LR, GFDL-ESM2M, MIROC-ESM-CHEM, NorESM1-M). All these scenarios assume the full CO_2 fertilization effect. In order to span the full space of uncertainty, the most severe scenario, RCP 8.5 from HadGEM2-ES, is also considered without CO_2 fertilization. Exogenous crop yield impacts from these climate scenarios are developed using data discussed in Leclère et al (2014) developed for the Agricultural Model Inter-Comparison Project.

Exogenous yield projections for crops and grassland vary significantly across regions and RCPs, especially in the United States. For corn, this projected change ranges less than 20% under RCPs 2.6, 4.5, and 6.0 and approximately 40% to 48% for RCP 8.5 (with and without CO_2 fertilization, respectively). For most other regions, projected yield changes follow similar trends, although the net effect is smaller than for the United States. In Canada and SAS, corn yields are projected to increase for RCPs 2.6, 4.5, and 6.0. Projected soybean yields show similar trends overall, although they are slightly less sensitive than corn overall to projected climate change impacts (especially for regions outside of the United States).

Projected wheat yields show the largest net change and the greatest variability across regions and RCPs. Exogenous yield projections are extremely sensitive in the United States, decreasing by a range of 10% to 50% by 2050 in the United States relative to a no climate change future. Other regions, including Canada, East Asia, and Sub-Saharan Africa, show sharp declines as well. Other regions show near-zero or positive yield impacts for wheat, including

Europe (EUR), the Middle East and North Africa (MNA), and Latin America and the Caribbean (LAC).

Finally, grassland yields are projected to increase nearly unanimously across all regions and scenarios, with notable exceptions being Sub-Saharan Africa and Latin America. In general, these results suggest that grassland productivity could offset some of the lost productivity of primary livestock feed grains such as corn. As discussed in this section, these productivity changes result in significant projected land use changes within the United States in response to the various climate change scenarios. Figures A2-A5 show projected exogenous crop yield impacts using the EPIC model.

GLOBIOM Trade Specification:

GLOBIOM international trade representation is based on the spatial equilibrium modelling approach, where individual regions trade with each other based purely on cost competitiveness because goods are assumed to be homogenous (Takayama and Judge, 1971). The trade calibration method proposed by Jansson and Heckelei (2009) is applied to reconcile observed bilateral trade flows based on COMTRADE, regional net trade based on FAOSTAT, prices, and trading costs for the base year. The model includes both tariffs, from the MAcMap-HS6 database (Bouët et al., 2008), and transportation costs (Hummels, 2001), differentiated among products and trading partners. Finally, endogenous trade cost increasing with the size of trade flows is included, which allows to introduce some level of stickiness in the trading patterns across partners. This non-linear element is represented through a single product constant elasticity cost function for regions where trade flows exist in the base year.

For new trade flows, a quadratic trade cost function allows them to be created, and once these new trade flows have been created, the quadratic function is replaced by the constant

elasticity function in the subsequent period. Indeed, GLOBIOM is a recursively dynamic model, which solves in 10 year time steps. Solution of the current period determines the starting conditions for the next period. Trade cost function is always repositioned to the trade quantity from the preceding period, hence the unit trade cost at the level corresponding to the solution of the previous period will be the same as the initial unit trade cost. Thus, the increasing component represents the additional, temporary, cost directly related to the trade flow expansion. The trade cost elasticities have been calibrated to roughly reproduce recent trade flows developments. However, a perfect reproduction is not possible because the trade flows are driven also by other drivers, in particular international trade agreements developments, which are considered constant in the model.

There is a large diversity in approaches how global economic models represent the international trade (von Lampe et al. 2014). Most of the partial equilibrium models consider a non-spatial equilibrium, with homogenous goods assumption, and exogenous, fixed, trade cost. On the other hand, the general equilibrium models typically make the assumption of heterogeneous goods, where an Armington elasticity determines the flexibility in substitution between domestically produced and imported products. The world pool market with homogenous goods will typically lead to much more flexibility in international trade adjustments to a climate shock than the Armington spatial equilibrium representation (Stehfest et al. 2013). The GLOBIOM trade representation stands between these two approaches, where the increasing trade cost component stabilizes the trade patterns similarly to the Armington approach, and at the same time the homogenous goods assumption brings the trade specification closer to the world pool market approach of the other partial equilibrium models. According to the detailed model intercomparison presented by Nelson et al. (2014), GLOBIOM performs on many aspects very

similar to the other well-established models. However, its trade specification and the underlying assumptions about future market integration by 2050, position it among the models with the strongest responsiveness of trade to climate change impacts.

Still, for long term projections of climate change impacts, in particular if adaptation, including trade expansion, can be planned, the system responsiveness to the climate shocks can be further enhanced. To model this option, we include a scenario, where the increasing trade cost element of the GLOBIOM trade specification, is reduced to almost zero. Specifically, all baseline trade elasticities in GLOBIOM are multiplied by 20, and the slope of the quadratic function is divided by four. This design brings the trade cost representation closer to other partial equilibrium models, which consider the trade cost fixed.

Livestock Sector Impacts from Base Scenario Design

Table 3 shows livestock commodity impacts, including production and prices. For each region and trade scenario combination, these reported values represent the percentage difference from the no climate change baseline in 2050, averaged over all climate change scenarios. Most livestock commodities summarized here see lower total production and higher prices, with average price impacts for pork, chicken, and eggs exceeding 10% under base trade assumptions. Production and price impacts for milk are smaller but are still negative and positive, respectively. However, beef production expands, and prices are lower, on average, relative to the no climate change baseline across these impacts scenarios. The strong and positive yield effect on US grasslands under the climate change scenarios considered helps maintain productivity of US cattle production systems, which tempers global market and price effects of climate change and reduced feed availability for livestock specifically. In these scenarios, US hog and poultry

production systems are more vulnerable to reduced feed grain availability and higher input costs; hence, production declines.

Production Impacts				
**Trade**	**Base Trade (T0)**		**Expanded Trade (T2)**	
Regional Extent of Climate Impacts	**USA**	**WLD**	**USA**	**WLD**
**Bovine Meat**	3.1%	−0.8%	5.3%	1.1%
**Pork Meat**	−5.1%	−4.3%	−14.9%	−15.4%
**Poultry Meat**	−3.0%	−3.7%	−6.4%	−6.4%
**Eggs**	−2.4%	−2.8%	−3.2%	−3.4%
**Milk**	−1.5%	−2.2%	−5.2%	−21.4%
**Price Impacts**				
	**Base Trade (T0)**		**Expanded Trade (T2)**	
Regional Extent of Climate Impacts	**USA**	**WLD**	**USA**	**WLD**
**Bovine Meat**	−4.2%	−4.2%	−4.8%	−5.0%
**Pork Meat**	11.7%	13.6%	1.4%	−0.4%
**Poultry Meat**	12.0%	13.6%	12.3%	13.2%
**Eggs**	11.5%	13.1%	11.9%	12.8%
**Milk**	2.1%	2.3%	0.9%	-2.7%

Table 3: Average livestock commodity impacts for the US relative to the no climate change baseline across RCP and GCM scenarios (2050 simulation period). Changes in USA production (top) and price (bottom) for major livestock products are presented for scenarios where climate impacts are applied only to the USA (USA, on the left of each of the four sets of results) and to the entire world (WLD).

Similar relationships between market power and net differences between global and domestic impacts scenarios shown for crop commodities in Figure 4 in the main body of the paper are seen in projected livestock sector impacts. Results presented in Figure 5 indicate that for livestock commodities, the commodity's share of global exports is a possible indicator of the potential difference between impacts in domestic and global scenarios. US-produced bovine

meat shows the largest relative shift in net production and price impacts between the domestic and global scenarios. Although US beef has commanded a significant share of total global production recently (18%), the total export share has been smaller (6%) (FAO 2016). US pork and poultry meat exports command a higher global market share; thus, price and production changes are similar between domestic and global impacts scenarios.

However, it is important to note that the US bovine meat system is boosted by climate change productivity shocks on grassland, and this factors into the net change in production and price impacts relative to the no climate change baseline. Under domestic impacts scenarios, US production increases slightly (on average) relative to the no climate change baseline. However, under global scenarios, beef production sees a modest decrease relative to the baseline as less land shifts into grassland in the United States and crop production expands slightly relative to the USA scenarios as the United States seeks to meet global grain demand with reduced productivity in all regions. Thus, while market share likely plays a role in explaining the net change in price and production impacts for US beef when moving from domestic to global impacts scenarios, part of this shift is likely driven by the general supply response present in US beef production systems driven by improved forage productivity.

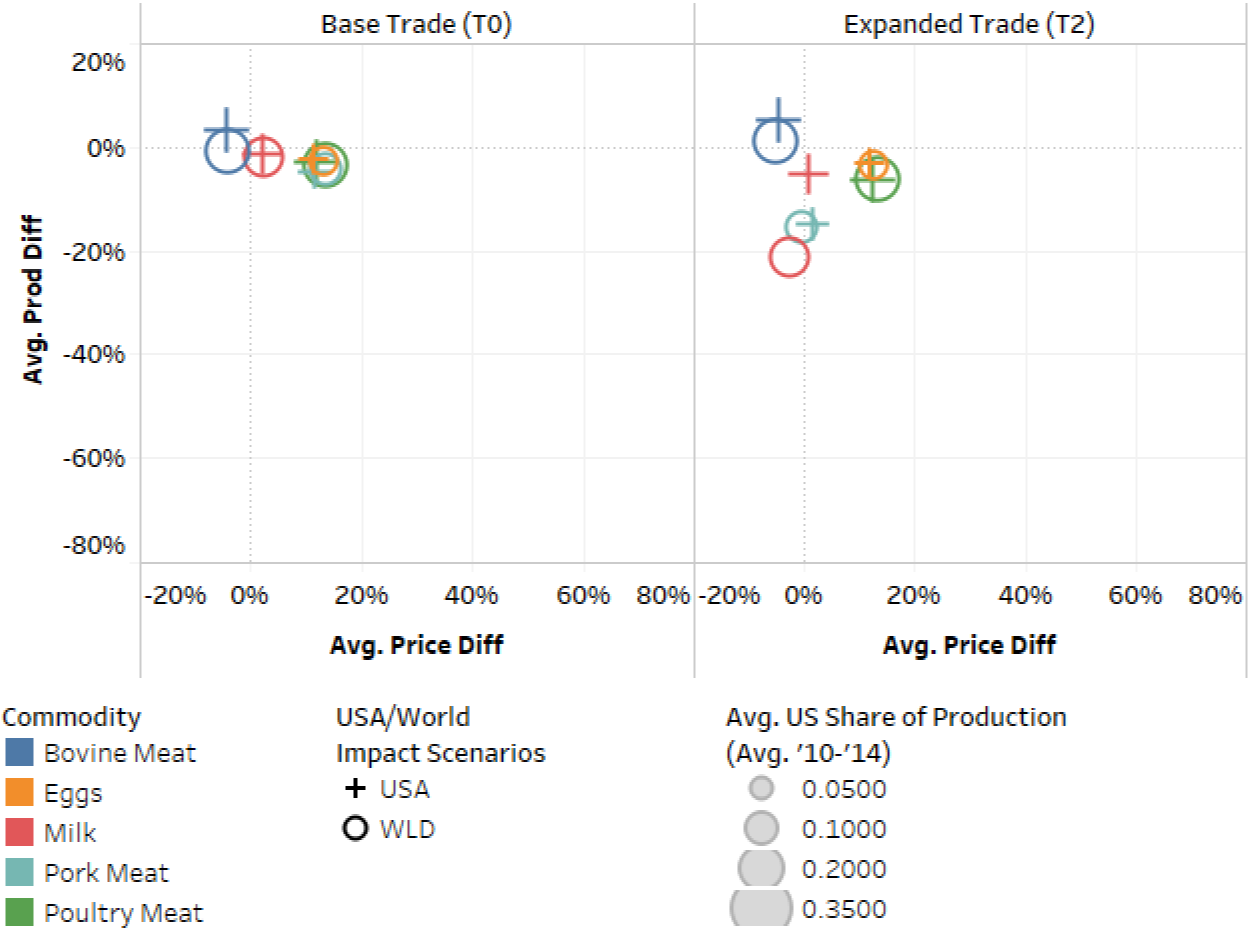

Figure A1: Average production and price impacts in the US for key livestock commodities across RCP and GCM scenarios and relative to recent US market share. The left panel figure includes average impacts for the scenarios with base model trade assumptions (T0) and the right-hand side shows average impacts with expanded trade (T2). Domestic (USA) and global climate impacts scenarios (WLD) are differentiated by symbol. The size of each shape corresponds to a metric related to market power. The size of the symbol corresponds to the US share of observed global production from 2010 to 2014 for the specific commodity group.

Robustness Checks—Crop Model Inputs and Trade Assumptions

In addition to the sensitivity analysis summarized in the main body of the manuscript, we conducted robustness checks involving additional scenarios around key model inputs and alternative trade assumptions. These robustness checks are designed to evaluate the relative difference in domestic vs. global zone of impact for situations in which crop yield impacts are

less severe (and positive in many cases) and for intensive margin trade cost changes (reduced tariffs). These additional scenarios allow us to assess whether the basic storylines of the base scenario design and implications of zone of impact considerations hold with alternative crop yield projections and trade assumptions. Information on scenario design parameters and key takeaways from these additional scenarios are summarized below.

Crop Model Yield Projections

The first robustness check was applied to projected yield changes from crop models. Recognizing the inherent uncertainty in biophysical crop modeling (as summarized in the paper), we replicated our full scenario design using a second crop model, the Lund-Postdam-Jena managed Land model (LPJmL). Specifically, we incorporated projected crop yield impacts across the set of RCP and GCM combinations already summarized using the LPJmL model. Data were obtained from the AgMIP scenarios (Rosenzweig et al. 2014) and incorporated into the GLOBIOM modeling framework as exogenous future yield change assumptions, like the EPIC crop model projections. The LPJmL model was chosen because it offers a relatively optimistic perspective on future crop yield changes, due in part to a large assumed CO_2 fertilization effect, so projections of exogenous crop yield changes show greater potential benefits than the projections used for the basis of this manuscript. By comparing EPIC and LPJmL scenarios as a robustness check, we can discern whether using moderate (or positive) projections of climate change-induced crop productivity changes will induce logically similar (though directionally different) results when comparing domestic and global zone of impact scenarios.

Figures A6-A9 represent exogenous crop yield change estimates from LPJmL for the 2050 simulation period plotted against EPIC projections for a direct comparison. All yield changes in these figures represent the percent difference from projected yields with no climate

change. Relative to EPIC simulations, LPJmL projections show more positive impacts for many crop and region combinations. For corn and wheat, LPJmL projected yield changes increase in many regions, including more vulnerable developing regions, whereas EPIC shows declining yields for most GCM and RCP combinations. Soybean yield change projections are positive and large for almost all crop and region combinations. Projected grassland yield changes, like EPIC projections, are mostly positive for RCP and GCM combinations, but are substantially larger. In total, the LPJmL projections result in a much more positive outlook for agricultural productivity in the future relative to EPIC, especially in regions with less developed agricultural sectors such as Sub-Saharan Africa, Latin America and the Caribbean, and Southeast Asia.

Figures A10-A15 offer a direct comparison of projected impact measures from GLOBIOM simulations between EPIC and LPJmL scenarios and for trade scenarios T0 and T2. There are a few key takeaways from our LPJmL scenarios worth noting. First, projected impacts are both less extreme, with smaller productivity and price impacts overall. In many cases, impacts are positive (e.g., increased yields and production) as projected exogenous yields changes higher relative to the no climate change scenario for many crop-region combinations. Because several regions exhibit increased yields, net impacts are moderated in U.S. systems, with the only meaningful price impacts occurring for the RCP 8.5 without CO_2 fertilization scenario.

U.S. corn systems show smaller impacts and demonstrate a tighter distribution for the LPJmL scenarios than for EPIC-derived simulations (Figure A10 and Figure A11). Most LPJmL scenarios, including RCP 8.5 without CO_2 fertilization, show impacts that fall within the mid-range of the distribution of impacts from EPIC scenarios, but the direction of these impacts varies in some cases (e.g., less U.S. area devoted to corn production given the modest global

yield response). Soybean impacts are mostly positive under LPJmL, with increased yields and lower prices for most scenarios except the RCP 8.5 without CO_2 fertilization.

U.S. wheat impacts are different in direction, magnitude, and relative variation when compared to the EPIC scenarios (Figure A13). U.S. wheat area expands substantially, and price impacts are close to zero or negative relative to the baseline (no CC) scenario, and with less variability across scenarios overall relative to EPIC simulations. Like corn and soybeans, the greatest price impact occurs for the RCP 8.5 without CO_2 fertilization scenario.

Second, while overall impacts are modest or differ in sign for LPJmL scenarios, we find a consistent storyline between domestic and global zone of impact scenarios for different U.S. crop systems. Corn and soybean systems see small relative changes in projected impacts relative to the no climate change baseline when comparing domestic and global zone of impact scenarios. These crop groups also show smaller relative trade adjustments overall between the T0 and T2 scenarios with LPJmL exogenous crop yield inputs. Smaller variation across RCPs and GCMs, and smaller net differences between zone of impact and trade scenarios are driven by shifting productivity growth outside of the U.S. For instance, corn productivity greatly increases for many regions outside of the U.S., so net price impacts are actually smaller and the U.S. reduces its area share of corn under the global zone of impact scenarios. With expanded trade, U.S. corn production decreases slightly relative to the T0 scenarios and price impacts are larger, suggesting a general shift of corn production and expanded trade outside of the U.S. where relative yields are increasing. Furthermore, key impact metrics for corn show larger relative differences between domestic and global zone of impact scenarios under T2 (and relative to T0), which further emphasizes the importance of interactions between climate change and trade considerations.

Soybean systems see increased yields for almost all region and RCP/GCM combinations under the LPJmL scenarios, unlike EPIC projections, which show mostly negative impacts (especially for soybean producing regions). When comparing domestic and global zone of impact scenarios, we find results that are opposite in sign from the EPIC simulations, but consistent in logic. Higher yields globally under the global zone of impact scenarios relaxes pressure on U.S. soybean systems, which reduces yields and production, with a small (<5%) mean decrease in the price impact as well (averaged across all RCPs and GCMs). Similar results for soybean systems are seen for T2.

Crop groups for which the US maintains a smaller total net market share, including wheat, also show net differences across alternative zone of impact and trade scenarios. Wheat shows similar directional changes to soybean systems when comparing domestic and global zone of impact scenarios, though the relative mean impact difference in prices is larger for wheat than for soybean or corn systems, which is consistent with our findings under the EPIC simulations. However, the relative change in wheat impact metrics between domestic and global impact scenarios is less for the LPJmL scenarios than for EPIC, especially under T2. Thus, while there are differences in our impact metrics for smaller market share commodity groups for the LPJmL scenarios across zone of impact and trade considerations, these differences often vary in both direction and magnitude relative to the EPIC scenarios and in general show less sensitivity to exogenous scenario assumptions.

The policy implication of this result is that while zone of impact and trade considerations do still matter when projecting impacts from less extreme scenarios, they matter far less than for impact assessments with more pessimistic exogenous productivity shocks. That is, while this manuscript argues that domestic impact assessments which ignore connections to global markets

and possible trade adjustments may over- or under-project climate change impacts, the magnitude of this bias is likely smaller when anticipated productivity changes are close to zero or positive.

Alternative Trade Specification Adjustments—Reduced Tariffs

The second set of robustness checks were applied to the alternative trade scenario specification. The T2 scenario design was developed to assess possible market outcomes and differences in impact measures for U.S. systems under extensive margin expansion in trade globally. That is, T2 scenarios were designed to assess domestic market and production impacts under climate change scenarios in which if non-tariff and non-transportation barrier costs to trade were reduced, markets were more fully integrated, and new trade flows emerged. This base scenario design focuses on the extensive margin for trade scenarios since heterogeneity in projected yield impacts under climate change across modeled regions could result in shifting comparative advantages in the production of individual commodities. By reducing the costs of establishing new trade flows, the goal was to assess how possible changes in bilateral trade might emerge with evolving comparative advantage in production as influenced by the alternative climate scenarios, all while holding current trade-related policies and transportation costs constant.

To assess the relative importance of reducing extensive margin trade costs on impacts metrics, we perform a second series of robustness checks around intensive margin trade cost assumptions. Specifically, we developed an alternative trade specification (T3) that holds extensive margin trade costs (quadratic functions) at their base level, but then incentivizes intensive margin trade adjustments by completely phasing out tariffs by 2030. This reduction in tariffs decreases trade costs overall and can lead to increased trade flows in existing markets.

Under T3, cost functions for establishing new trade flows are held at baseline levels (unlike T2 where the slopes of these cost functions are lowered).

Figures A16-18 provide a comparison of results for the T0, T2, and T3 trade scenarios, using the EPIC crop model inputs. We find that reducing existing tariffs has a meaningful effect on net impacts by reducing trade costs, which lowers overall market price impacts and shifts the distribution of other impact metrics. With reduced tariffs, there is less variability in corn production impacts, and price changes are smaller. U.S. soybean area changes relative to the no climate change baseline are larger under T3 than T0, with a smaller decrease in total production as the U.S. expands soybean exports under T3 relative to T0 climate scenarios. Soybean price impacts are also smaller for T3 than for T0 as trade costs are lower. Wheat impacts are also less extreme for T3 than for T0.

Thus, in general, results under T3 show a similar story to T0 impacts, but with reduced market price impacts and tighter distributions. The relative difference in impacts for corn and soybean systems between domestic and global zone of impact scenarios are similar for T3 than for T0. For wheat systems, the relative change in impacts from domestic global is smaller under T3 than under T0 and T2 but directionally consistent to the T0 narrative. The T3 trade scenario structure introduces fewer relative changes in U.S. wheat systems across various climate scenarios relative to the extensive margin (T2) trade adjustments. The small relative changes under T3 are due to low relative tariff-related trade costs for U.S. wheat exports, as only one bilateral trading partner (Japan) imposes a tariff on U.S. wheat in the 2050 simulation period of our T0 simulations. Thus, while changes in tariff-related trade costs have important net market effects (lower prices), these changes have minimal impact on U.S. wheat systems relative to the T0 scenarios.

The implication of this result is that for crop groups with a relatively high baseline market share and tariff costs, relative differences between domestic and global zone of impact scenarios are consistent across all trade scenarios (T0, T2, and T3). Crops with less market share are more sensitive to zone of impact and extensive margin trade assumptions (T2), but if baseline tariff costs are low, then these relative scenario differences are minimized under T3 in comparison to T2 and T0 scenarios. Thus, while our trade scenario sensitivities validate our zone of impact narrative for high market share crops, these results indicate that structure of crop specific trade costs under the baseline is another important determinant of whether domestic-focused impact assessments that do not account for global market changes bias impact results.

SUPPLEMENTAL REFERENCES

Bouët, A., Decreux Y., Fontagné L., Jean S., Laborde, D. 2008. Assessing applied protection across the world, *Review of International Economics* 16(5), February, 850-863.

Hummels, David. "Toward a Geography of Trade Costs." (2001).

JANSSON, T., and T. HECKELEI, (2009): A new estimator for trade costs and its small sample properties. Economic Modelling 26(2): 489-498.

Nelson G, Valin H, Sands RD, Havlik P, Ahammad H, Deryng D, Elliot J, Fujimori S, Hasegawa T, Heyhoe E, Kyle P, Von Lampe M, Lotze-Campen H, Mason d'Croz D, van Meijl H, van der Mensbrugghe D, Müller C, Popp A, Robertson R, Robinson S, Schmid E, Schmitz C, Tabeau A, and Willenbockel D 2014 Climate change effects on agriculture: Economic responses to biophysical shocks. *Proc. Natl Acad. Sci.* 111(9) 3274-3279

Rosenzweig C, Elliott J, Deryng D, Ruane AC, Müller C, Arneth A, Boote KJ, Folberth C, Glotter M, Khabarov N, Neumann K, Piontek F, Pugh TAM, Schmid E, Stehfest E, Yang H, and Jones JW 2014 Assessing agricultural risks of climate change in the 21st century in a global gridded crop model intercomparison. *Proc. Natl. Acad. Sci.* 111(9) 3268-3273, doi:10.1073/pnas.1222463110

Stehfest, Elke, et al. "Options to reduce the environmental effects of livestock production–comparison of two economic models." *Agricultural Systems* 114 (2013): 38-53.

Takayama T and Judge, G G 1971 *Spatial and Temporal Price and Allocation Models* (Amsterdam: North-Holland)

Nelson, G. C., et al. (2014) "Climate change effects on agriculture: Economic responses to biophysical shocks." Proceedings of the National Academy of Sciences. 111(9): 3274-3279.

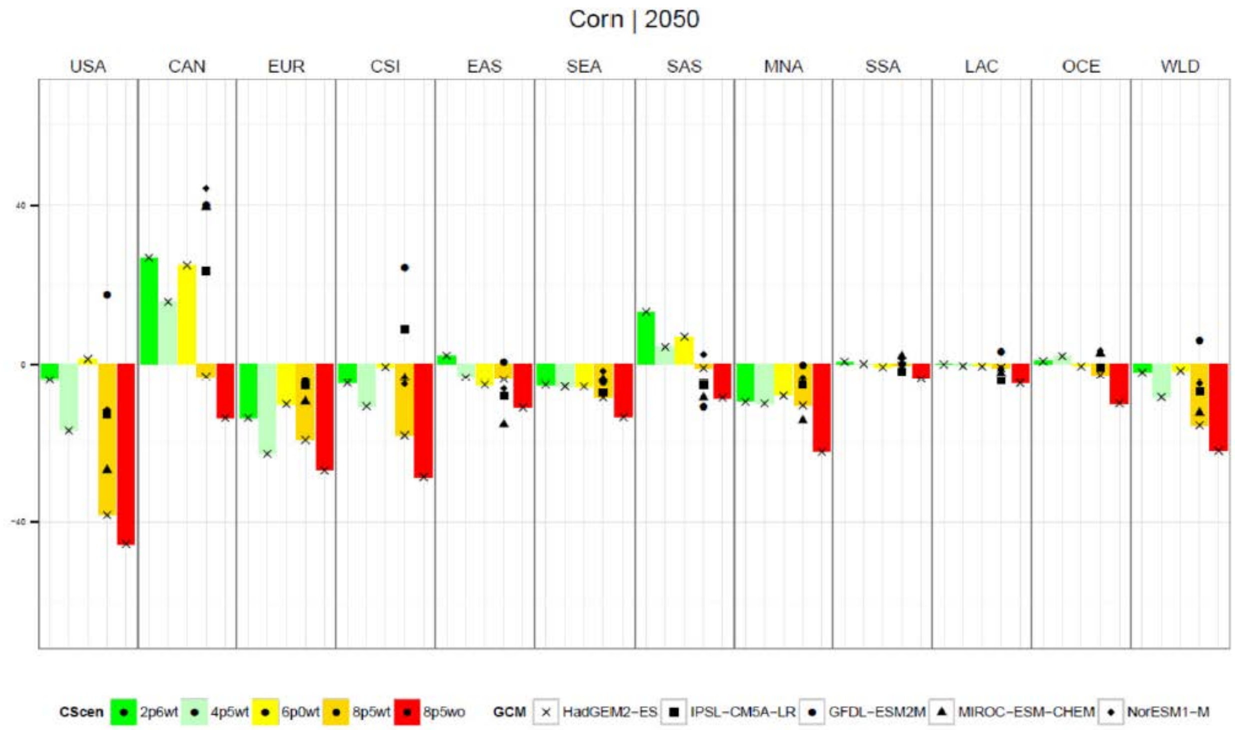

Figure A2: EPIC projected exogenous yield changes for corn production by major producing region (% difference from baseline)

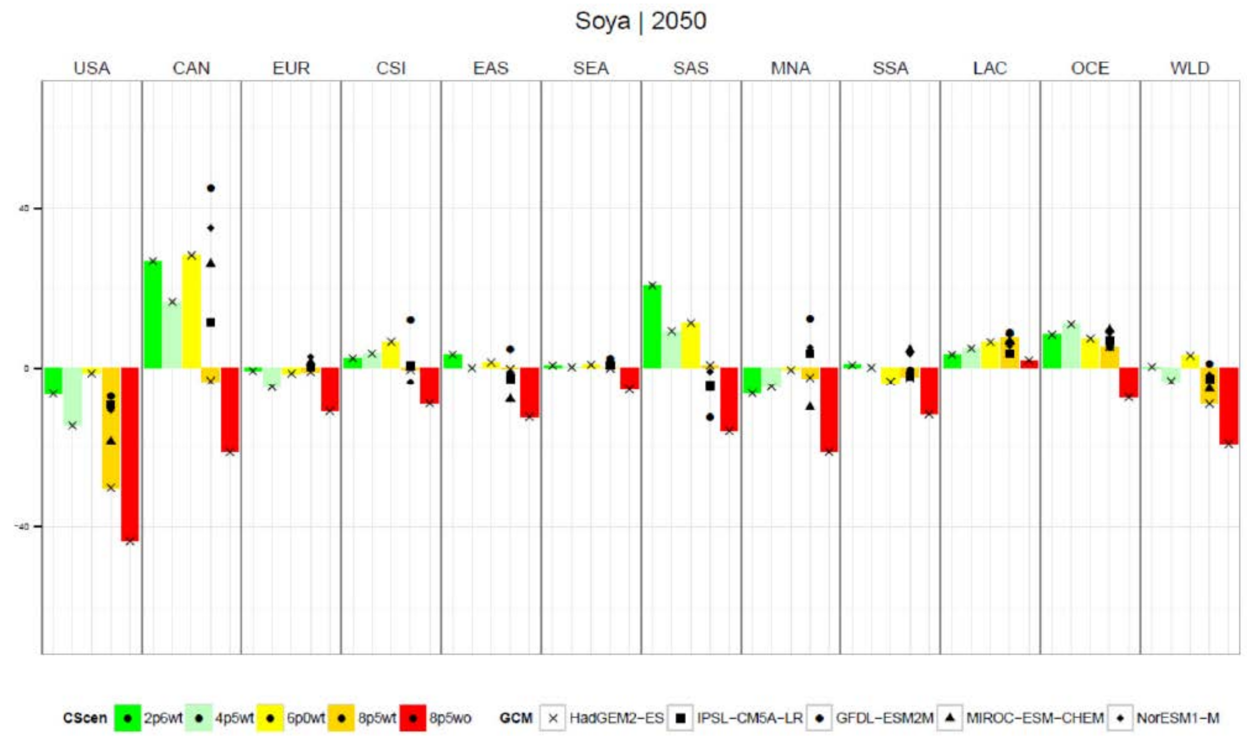

Figure A3: EPIC projected exogenous yield changes for soybean production by major producing region (% difference from baseline)

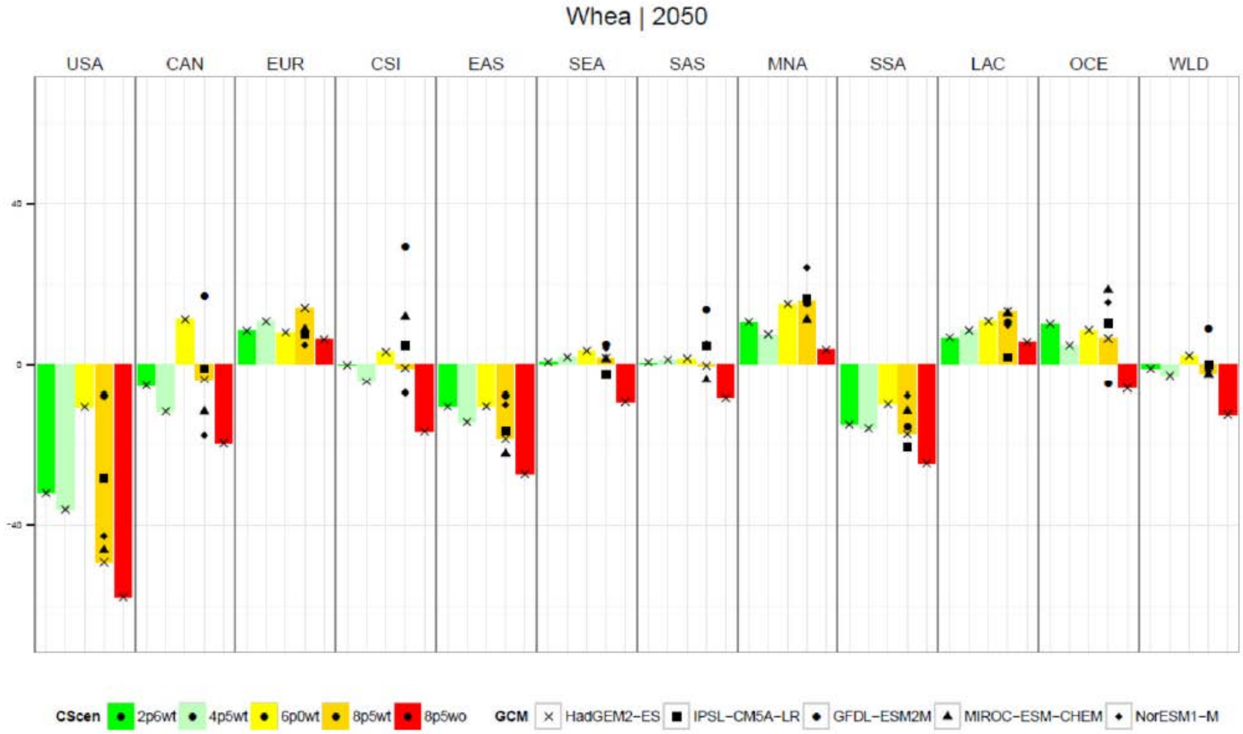

Figure A4: EPIC projected exogenous yield changes for wheat production by major producing region (% difference from baseline)

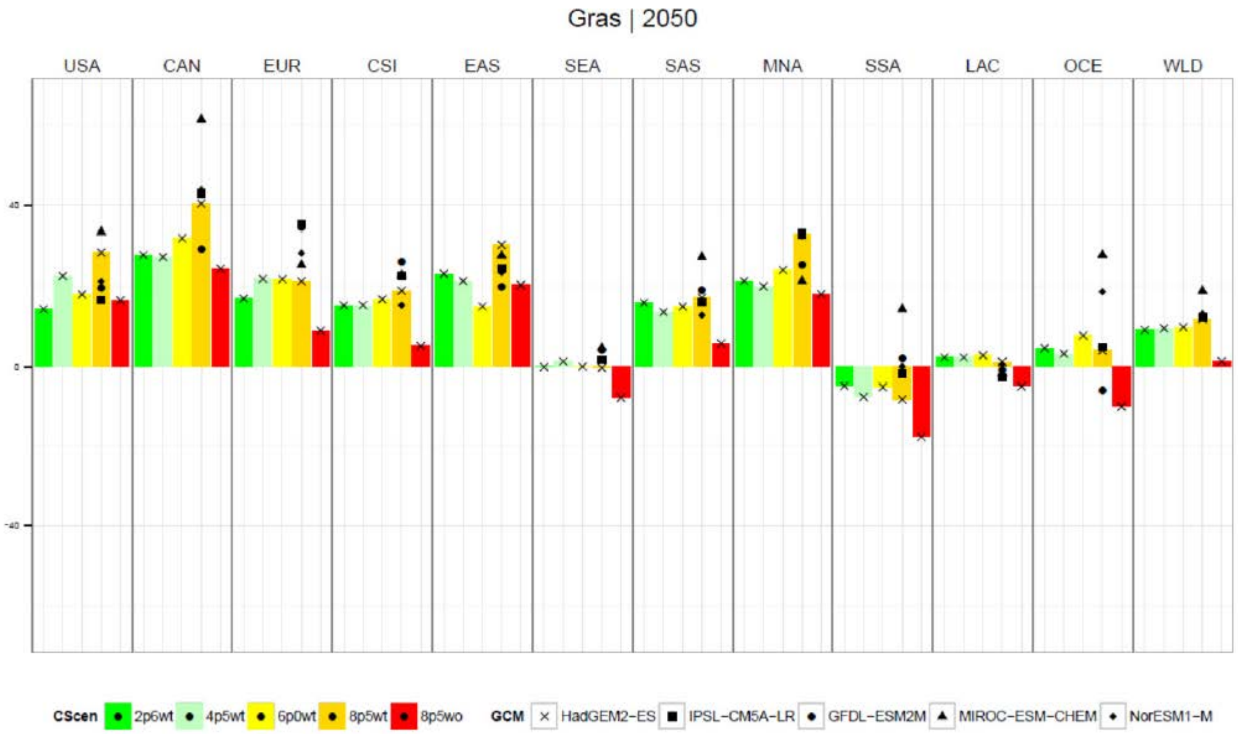

Figure A5: EPIC projected exogenous yield changes for grassland by major producing region (% difference from baseline)

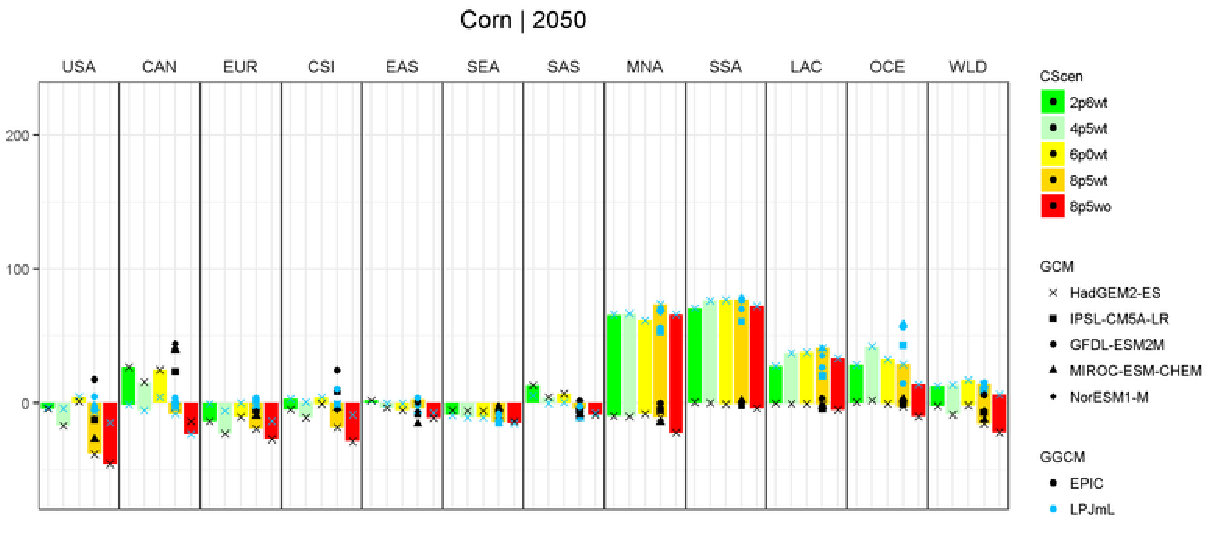

Figure A6: EPIC and LPJmL projected exogenous yield changes for corn production by major producing region (% difference from baseline)

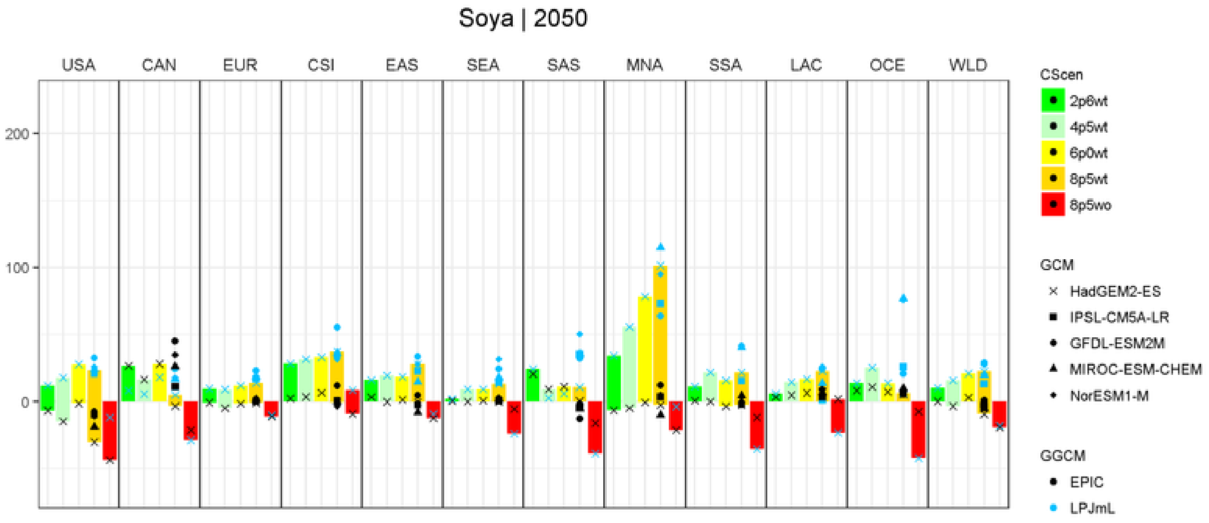

Figure A7: EPIC and LPJmL projected exogenous yield changes for soybean production by major producing region (% difference from baseline)

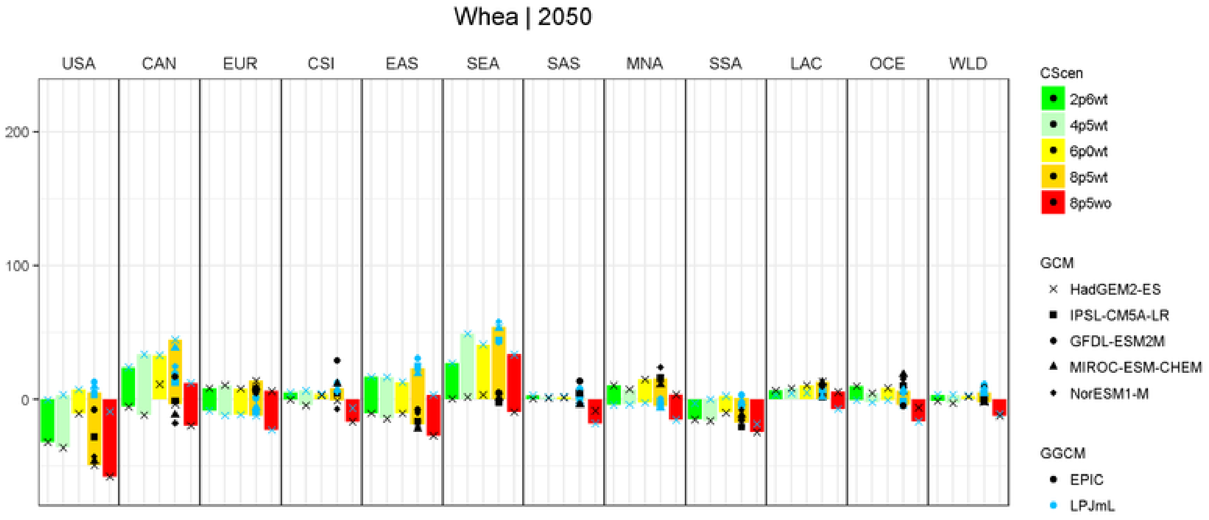

Figure A8: EPIC and LPJmL projected exogenous yield changes for wheat production by major producing region (% difference from baseline)

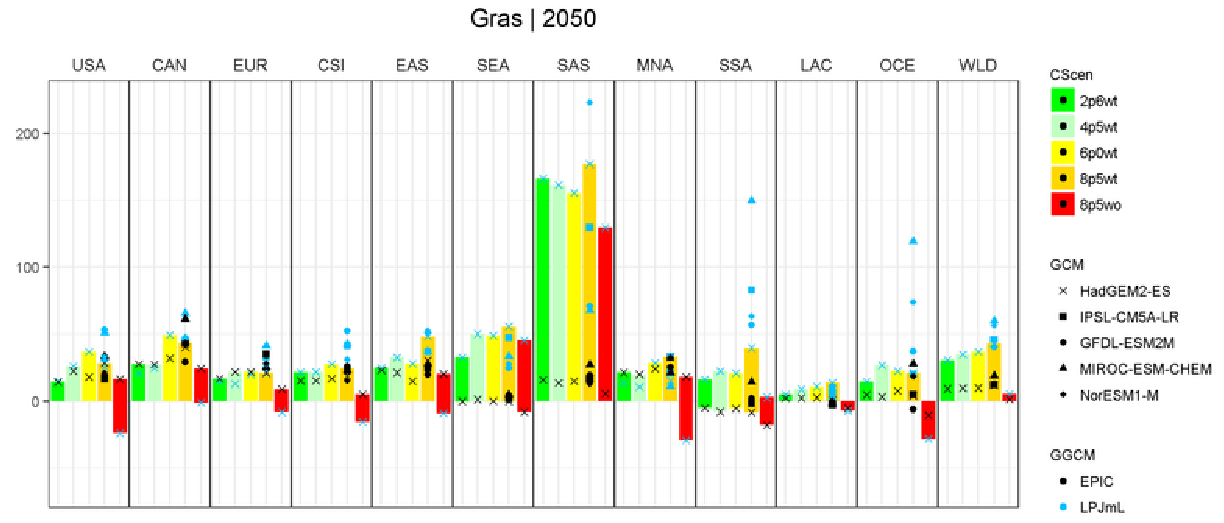

Figure A9: EPIC and LPJmL projected exogenous yield changes for grassland production by major producing region (% difference from baseline)

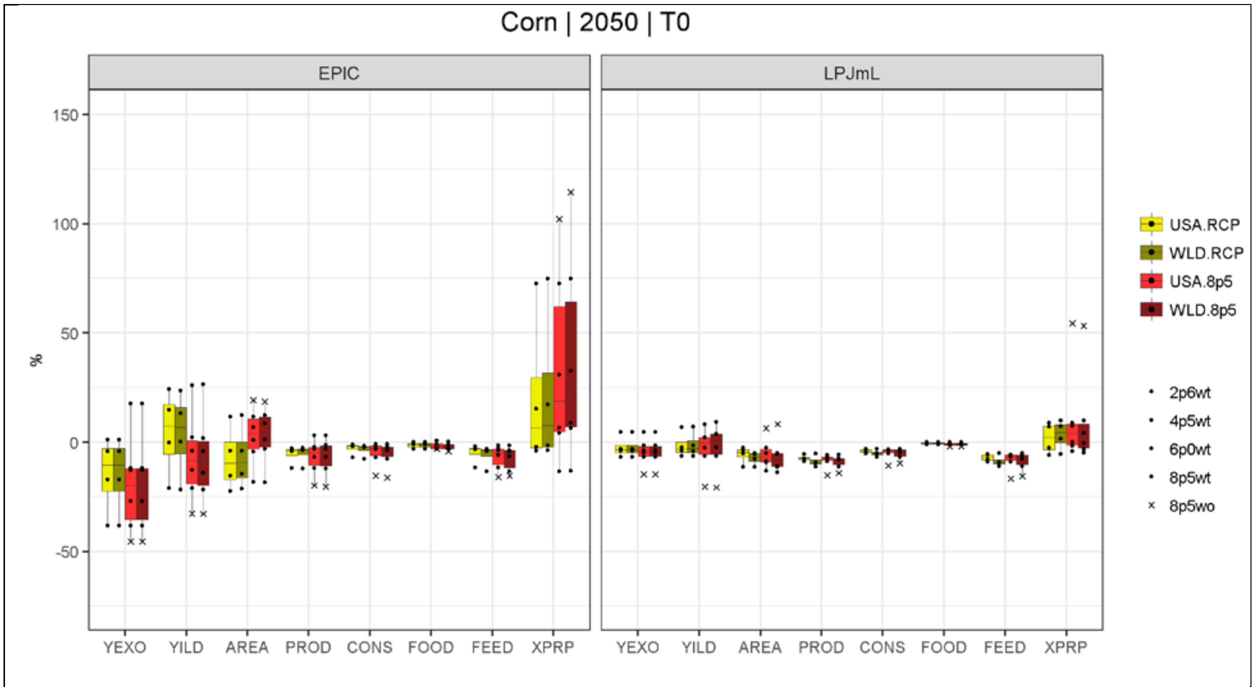

Figure A10: Projected climate impacts for US corn production systems across RCPs and GCMs when climate impacts are applied only domestically (USA) vs. climate impacts applied to the entire world (WLD). Values are provided for changes in exogenous yield inputs (YEXO), endogenous yield after market responses (YILD), crop area (AREA), production (PROD), total consumption (CONS) along with subcategories reflecting consumption for food (FOOD) and feed (FEED), and prices (XPRP). Yellow shaded bars show the interquartile range across all four RCPs with CO_2 fertilization for the scenarios where climate impacts are applied only domestically (USA). Green shaded bars show the interquartile range for the four RCPs with CO_2 fertilization for the scenarios where climate impacts are applied globally (WLD). Red bars show the interquartile range across 5 GCMs for RCP8.5 (with [8p5wt] and without [8p5wo] CO_2 fertilization for HadGEM ES2) for domestic-only impacts scenarios. Dark red bars show the interquartile range for GCMs for RCP8.5 (with and without CO2 fertilization) for global impacts scenarios. Both EPIC and LPJmL crop models are shown with baseline trade assumptions (T0).

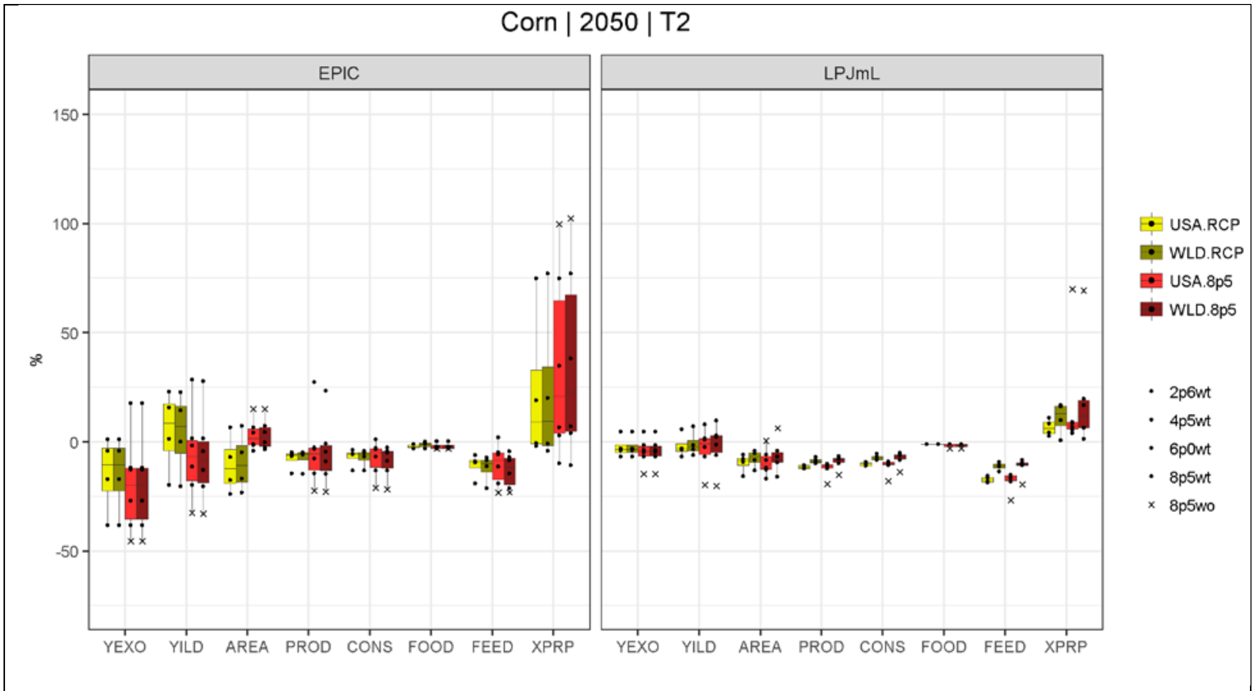

Figure A11: Projected climate impacts for US corn production systems across RCPs and GCMs when climate impacts are applied only domestically (USA) vs. climate impacts applied to the entire world (WLD). Values are provided for changes in exogenous yield inputs (YEXO), endogenous yield after market responses (YILD), crop area (AREA), production (PROD), total consumption (CONS) along with subcategories reflecting consumption for food (FOOD) and feed (FEED), and prices (XPRP). Yellow shaded bars show the interquartile range across all four RCPs with CO_2 fertilization for the scenarios where climate impacts are applied only domestically (USA). Green shaded bars show the interquartile range for the four RCPs with CO_2 fertilization for the scenarios where climate impacts are applied globally (WLD). Red bars show the interquartile range across 5 GCMs for RCP8.5 (with [8p5wt] and without [8p5wo] CO_2 fertilization for HadGEM ES2) for domestic-only impacts scenarios. Dark red bars show the interquartile range for GCMs for RCP8.5 (with and without CO2 fertilization) for global impacts scenarios. Both EPIC and LPJmL crop models are shown with expanded trade assumptions (T2).

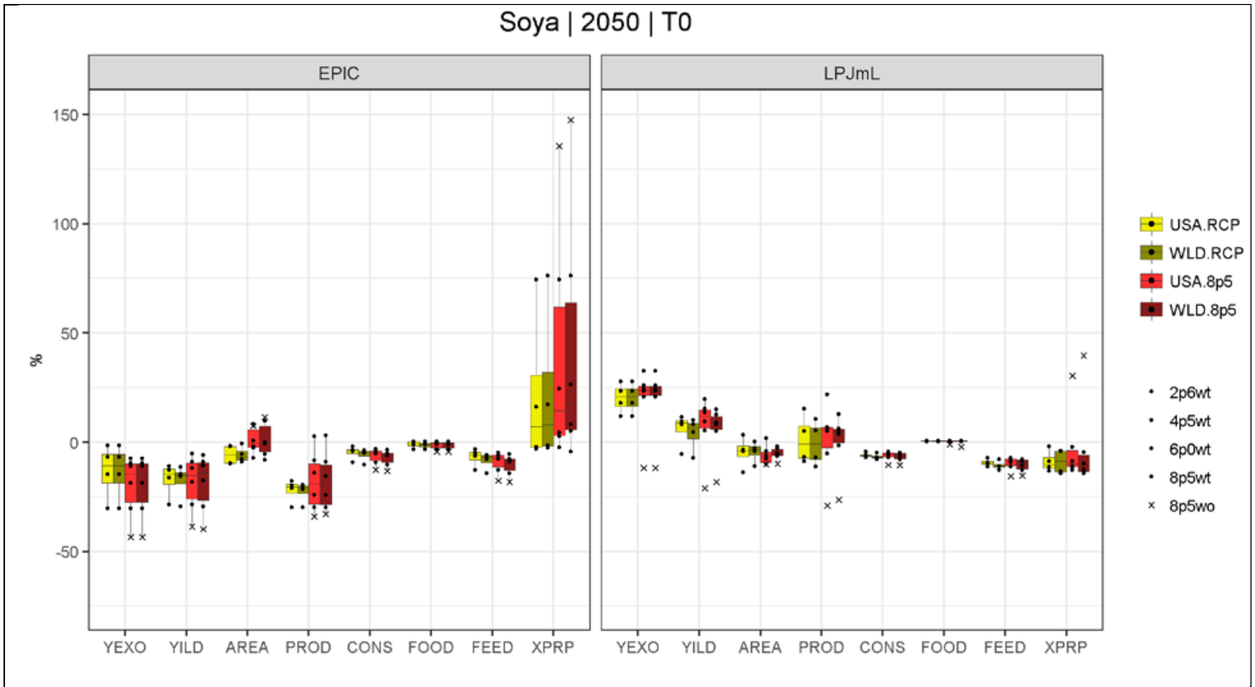

Figure A12: Projected climate impacts for US soybean production systems across RCPs and GCMs when climate impacts are applied only domestically (USA) vs. climate impacts applied to the entire world (WLD). Values are provided for changes in exogenous yield inputs (YEXO), endogenous yield after market responses (YILD), crop area (AREA), production (PROD), total consumption (CONS) along with subcategories reflecting consumption for food (FOOD) and feed (FEED), and prices (XPRP). Yellow shaded bars show the interquartile range across all four RCPs with CO_2 fertilization for the scenarios where climate impacts are applied only domestically (USA). Green shaded bars show the interquartile range for the four RCPs with CO_2 fertilization for the scenarios where climate impacts are applied globally (WLD). Red bars show the interquartile range across 5 GCMs for RCP8.5 (with [8p5wt] and without [8p5wo] CO_2 fertilization for HadGEM ES2) for domestic-only impacts scenarios. Dark red bars show the interquartile range for GCMs for RCP8.5 (with and without CO2 fertilization) for global impacts scenarios. Both EPIC and LPJmL crop models are shown with baseline trade assumptions (T0).

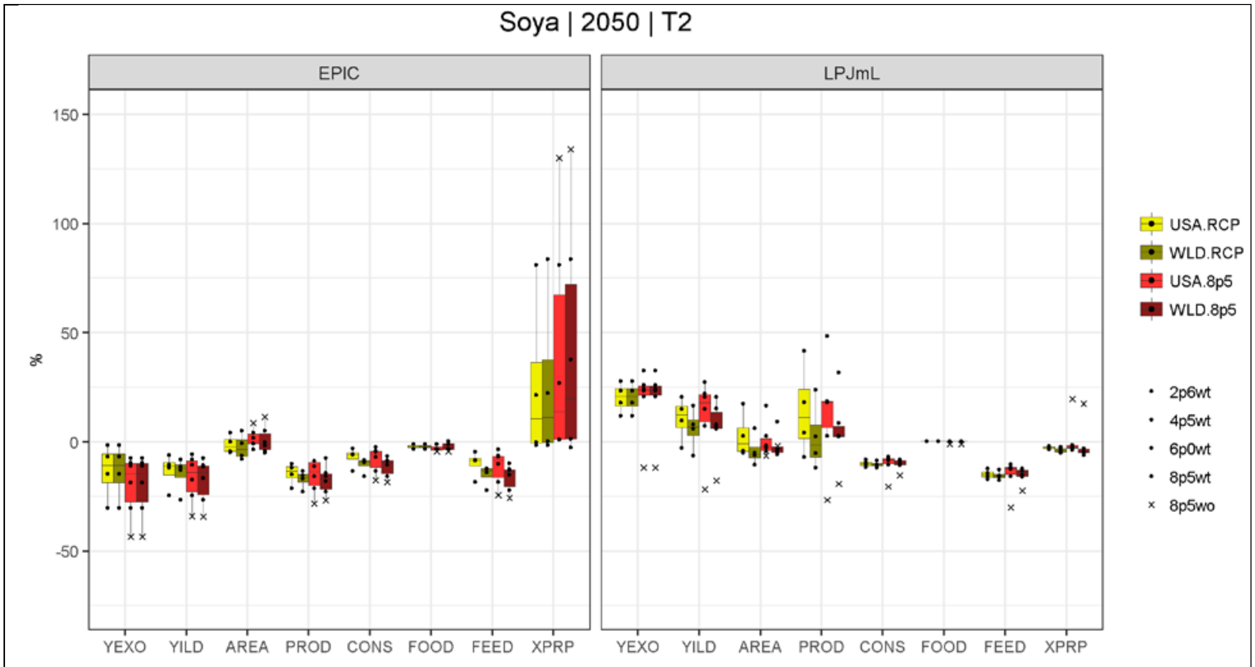

Figure A13: Projected climate impacts for US soybean production systems across RCPs and GCMs when climate impacts are applied only domestically (USA) vs. climate impacts applied to the entire world (WLD). Values are provided for changes in exogenous yield inputs (YEXO), endogenous yield after market responses (YILD), crop area (AREA), production (PROD), total consumption (CONS) along with subcategories reflecting consumption for food (FOOD) and feed (FEED), and prices (XPRP). Yellow shaded bars show the interquartile range across all four RCPs with CO_2 fertilization for the scenarios where climate impacts are applied only domestically (USA). Green shaded bars show the interquartile range for the four RCPs with CO_2 fertilization for the scenarios where climate impacts are applied globally (WLD). Red bars show the interquartile range across 5 GCMs for RCP8.5 (with [8p5wt] and without [8p5wo] CO_2 fertilization for HadGEM ES2) for domestic-only impacts scenarios. Dark red bars show the interquartile range for GCMs for RCP8.5 (with and without CO2 fertilization) for global impacts scenarios. Both EPIC and LPJmL crop models are shown with expanded trade assumptions (T2).

1

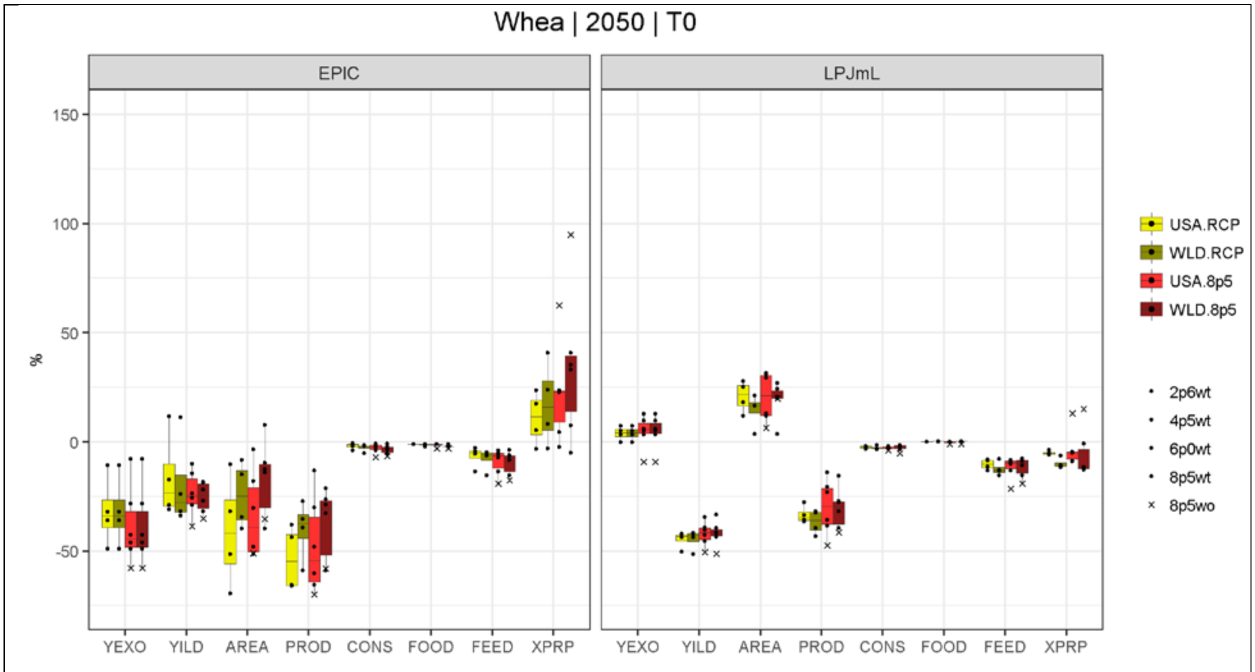

Figure A14: Projected climate impacts for US wheat production systems across RCPs and GCMs when climate impacts are applied only domestically (USA) vs. climate impacts applied to the entire world (WLD). Values are provided for changes in exogenous yield inputs (YEXO), endogenous yield after market responses (YILD), crop area (AREA), production (PROD), total consumption (CONS) along with subcategories reflecting consumption for food (FOOD) and feed (FEED), and prices (XPRP). Yellow shaded bars show the interquartile range across all four RCPs with CO_2 fertilization for the scenarios where climate impacts are applied only domestically (USA). Green shaded bars show the interquartile range for the four RCPs with CO_2 fertilization for the scenarios where climate impacts are applied globally (WLD). Red bars show the interquartile range across 5 GCMs for RCP8.5 (with [8p5wt] and without [8p5wo] CO_2 fertilization for HadGEM ES2) for domestic-only impacts scenarios. Dark red bars show the interquartile range for GCMs for RCP8.5 (with and without CO2 fertilization) for global impacts scenarios. Both EPIC and LPJmL crop models are shown with baseline trade assumptions (T0).

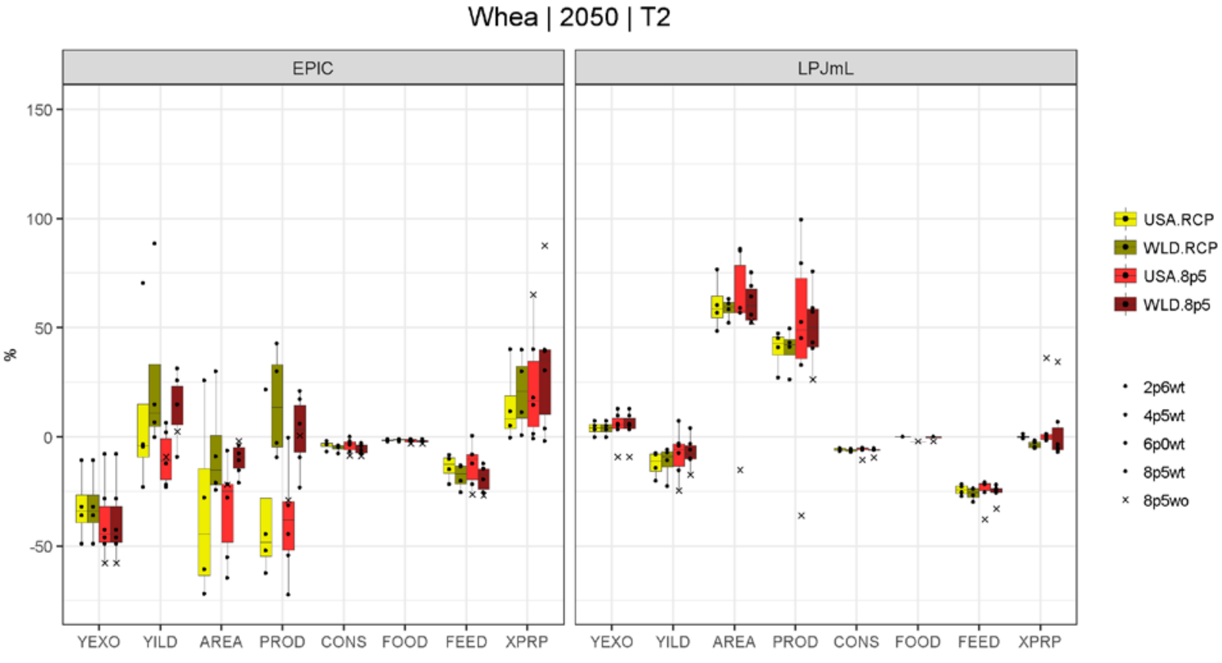

Figure A15: Projected climate impacts for US wheat production systems across RCPs and GCMs when climate impacts are applied only domestically (USA) vs. climate impacts applied to the entire world (WLD). Values are provided for changes in exogenous yield inputs (YEXO), endogenous yield after market responses (YILD), crop area (AREA), production (PROD), total consumption (CONS) along with subcategories reflecting consumption for food (FOOD) and feed (FEED), and prices (XPRP). Yellow shaded bars show the interquartile range across all four RCPs with CO_2 fertilization for the scenarios where climate impacts are applied only domestically (USA). Green shaded bars show the interquartile range for the four RCPs with CO_2 fertilization for the scenarios where climate impacts are applied globally (WLD). Red bars show the interquartile range across 5 GCMs for RCP8.5 (with [8p5wt] and without [8p5wo] CO_2 fertilization for HadGEM ES2) for domestic-only impacts scenarios. Dark red bars show the interquartile range for GCMs for RCP8.5 (with and without CO2 fertilization) for global impacts scenarios. Both EPIC and LPJmL crop models are shown with expanded trade assumptions (T2).

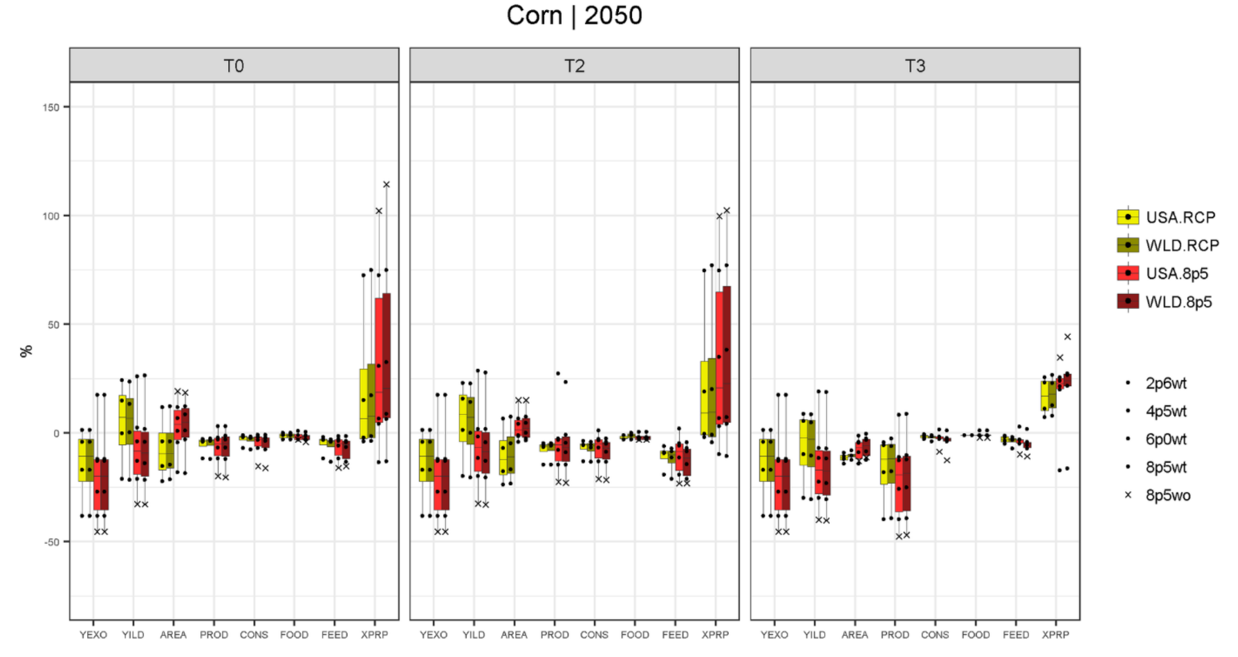

Figure A16: Projected climate impacts for US corn production systems across RCPs and GCMs when climate impacts are applied only domestically (USA) vs. climate impacts applied to the entire world (WLD). Values are provided for changes in exogenous yield inputs (YEXO), endogenous yield after market responses (YILD), crop area (AREA), production (PROD), total consumption (CONS) along with subcategories reflecting consumption for food (FOOD) and feed (FEED), and prices (XPRP). Yellow shaded bars show the interquartile range across all four RCPs with CO_2 fertilization for the scenarios where climate impacts are applied only domestically (USA). Green shaded bars show the interquartile range for the four RCPs with CO_2 fertilization for the scenarios where climate impacts are applied globally (WLD). Red bars show the interquartile range across 5 GCMs for RCP8.5 (with [8p5wt] and without [8p5wo] CO_2 fertilization for HadGEM ES2) for domestic-only impacts scenarios. Dark red bars show the interquartile range for GCMs for RCP8.5 (with and without CO2 fertilization) for global impacts scenarios. Results are shown for the EPIC crop model with baseline (T0), expanded trade (T2), and reduced tariff (T3) assumptions.

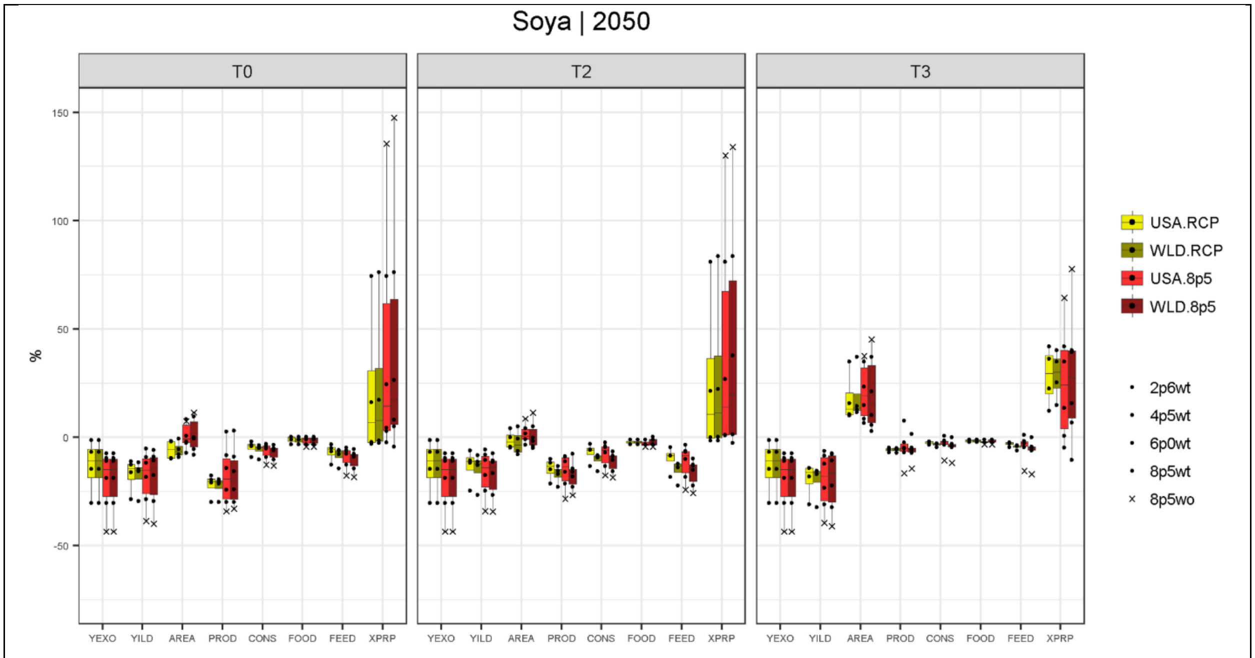

Figure A17: Projected climate impacts for US soybean production systems across RCPs and GCMs when climate impacts are applied only domestically (USA) vs. climate impacts applied to the entire world (WLD). Values are provided for changes in exogenous yield inputs (YEXO), endogenous yield after market responses (YILD), crop area (AREA), production (PROD), total consumption (CONS) along with subcategories reflecting consumption for food (FOOD) and feed (FEED), and prices (XPRP). Yellow shaded bars show the interquartile range across all four RCPs with CO_2 fertilization for the scenarios where climate impacts are applied only domestically (USA). Green shaded bars show the interquartile range for the four RCPs with CO_2 fertilization for the scenarios where climate impacts are applied globally (WLD). Red bars show the interquartile range across 5 GCMs for RCP8.5 (with [8p5wt] and without [8p5wo] CO_2 fertilization for HadGEM ES2) for domestic-only impacts scenarios. Dark red bars show the interquartile range for GCMs for RCP8.5 (with and without CO2 fertilization) for global impacts scenarios. Results are shown for the EPIC crop model with baseline (T0), expanded trade (T2), and reduced tariff (T3) assumptions.

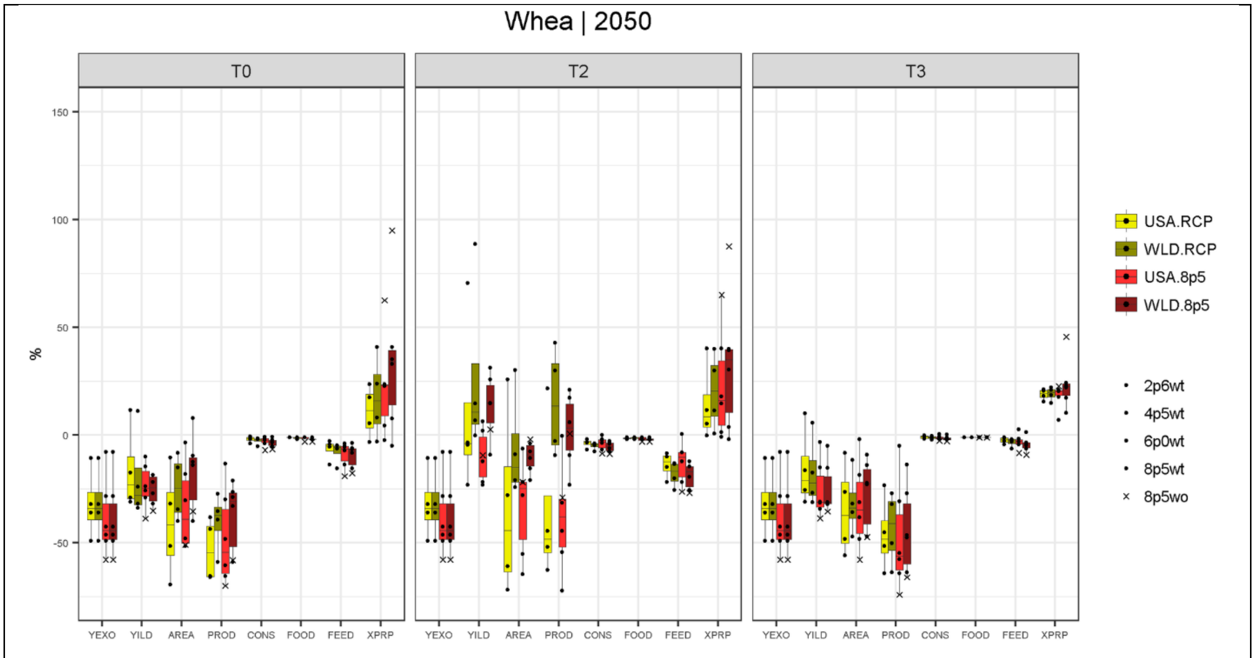

Figure A18: Projected climate impacts for US soybean production systems across RCPs and GCMs when climate impacts are applied only domestically (USA) vs. climate impacts applied to the entire world (WLD). Values are provided for changes in exogenous yield inputs (YEXO), endogenous yield after market responses (YILD), crop area (AREA), production (PROD), total consumption (CONS) along with subcategories reflecting consumption for food (FOOD) and feed (FEED), and prices (XPRP). Yellow shaded bars show the interquartile range across all four RCPs with CO_2 fertilization for the scenarios where climate impacts are applied only domestically (USA). Green shaded bars show the interquartile range for the four RCPs with CO_2 fertilization for the scenarios where climate impacts are applied globally (WLD). Red bars show the interquartile range across 5 GCMs for RCP8.5 (with [8p5wt] and without [8p5wo] CO_2 fertilization for HadGEM ES2) for domestic-only impacts scenarios. Dark red bars show the interquartile range for GCMs for RCP8.5 (with and without CO2 fertilization) for global impacts scenarios. Results are shown for the EPIC crop model with baseline (T0), expanded trade (T2), and reduced tariff (T3) assumptions.
